# Integrative taxonomy reveals three new taxa within the *Tylototriton asperrimus* complex (Caudata, Salamandridae) from Vietnam

**DOI:** 10.3897/zookeys.935.37138

**Published:** 2020-05-21

**Authors:** Marta Bernardes, Minh Duc Le, Truong Quang Nguyen, Cuong The Pham, Anh Van Pham, Tao Thien Nguyen, Dennis Rödder, Michael Bonkowski, Thomas Ziegler

**Affiliations:** 1 Cologne Zoo, Riehler Str. 173, 50735 Cologne, Germany Cologne Zoo Cologne Germany; 2 Terrestrial Ecology, Institute of Zoology, University of Cologne, Zülpicher Str. 47b, 50674 Cologne, Germany University of Cologne Cologne Germany; 3 Faculty of Environmental Sciences, University of Science, Vietnam National University, Hanoi, 334 Nguyen Trai Road, Hanoi, Vietnam University of Science Hanoi Vietnam; 4 Central Institute for Natural Resources and Environmental Studies, Vietnam National University, Hanoi, 19 Le Thanh Tong, Hanoi, Vietnam Vietnam National University Hanoi Vietnam; 5 Department of Herpetology, American Museum of Natural History, Central Park West at 79th Street, New York, New York 10024, USA American Museum of Natural History New York United States of America; 6 Institute of Ecology and Biological Resources, Vietnam Academy of Science and Technology, 18 Hoang Quoc Viet, Hanoi, Vietnam Graduate University of Science and Technology Hanoi Vietnam; 7 Graduate University of Science and Technology, Vietnam Academy of Science and Technology, 18 Hoang Quoc Viet, Cau Giay, Hanoi, Vietnam Vietnam Academy of Science and Technology Hanoi Vietnam; 8 Faculty of Natural Science and Technology, Tay Bac University, Quyet Tam Ward, Son La City, Son La Province, Vietnam Tay Bac University Son La City Vietnam; 9 Vietnam National Museum of Nature, 18 Hoang Quoc Viet St., Hanoi, Vietnam Vietnam National Museum of Nature Hanoi Vietnam; 10 Zoologisches Forschungsmuseum Alexander Koenig, Adenauerallee 160, 53113 Bonn, Germany Zoologisches Forschungsmuseum Alexander Koenig Bonn Germany

**Keywords:** conservation, crocodile newts, cryptic diversity, new records, South East Asia

## Abstract

The *Tylototriton asperrimus* complex from northern Vietnam is reviewed based on morphological comparisons and analysis of the mitochondrial marker NADH dehydrogenase subunit 2 (ND2). Based on molecular divergences, which were revealed to be higher than in other congeners, in concert with morphological differences, two new species and one subspecies are described herein: *Tylototriton pasmansi***sp. nov.** differs from *T. asperrimussensu stricto* by 3.2 to 3.6 % genetic divergence and a combination of distinct morphological characters, such as head slightly longer than wide, distinct mid-dorsal ridge, relatively wide distance between the eyes, tips of fingers reaching the eye when foreleg is laid forward, labial and gular folds present, central belly skin with tubercles shaped like transverse wrinkles and distinct, pointy to round rib nodules. The population of *T. pasmansi***sp. nov.** consists of two subclades, the nominotypic one occurring on the eastern side of the Da River (or Black River, including Hoa Binh and Phu Tho provinces), and another occurring on the western side (including Son La and Thanh Hoa provinces). These two subclades differ by 2.5 to 3.1 % genetic divergence and distinct morphological characters. The western subclade is herein described as *Tylototriton pasmansi obsti***ssp. nov.**, which differs from the nominotypic form by a wider head, longer and narrower snout, shorter femur length, and an overall less granulose skin, without an increased concentration of warts on the body sides.

A second new species, *Tylototriton sparreboomi***sp. nov.** is described from Lai Chau Province. It differs from *T. asperrimussensu stricto* by 4.1 to 4.2 % and from *Tylototriton pasmansi***sp. nov.** by 3.6 to 4.5 % genetic divergences as well as by a combination of distinct morphological characters, such as head longer than wide, tips of fingers reaching nostril when foreleg adpressed along head, rib nodules distinct, round and relatively enlarged, and wide distance between the eyes.

## Introduction

**Figure 1.**
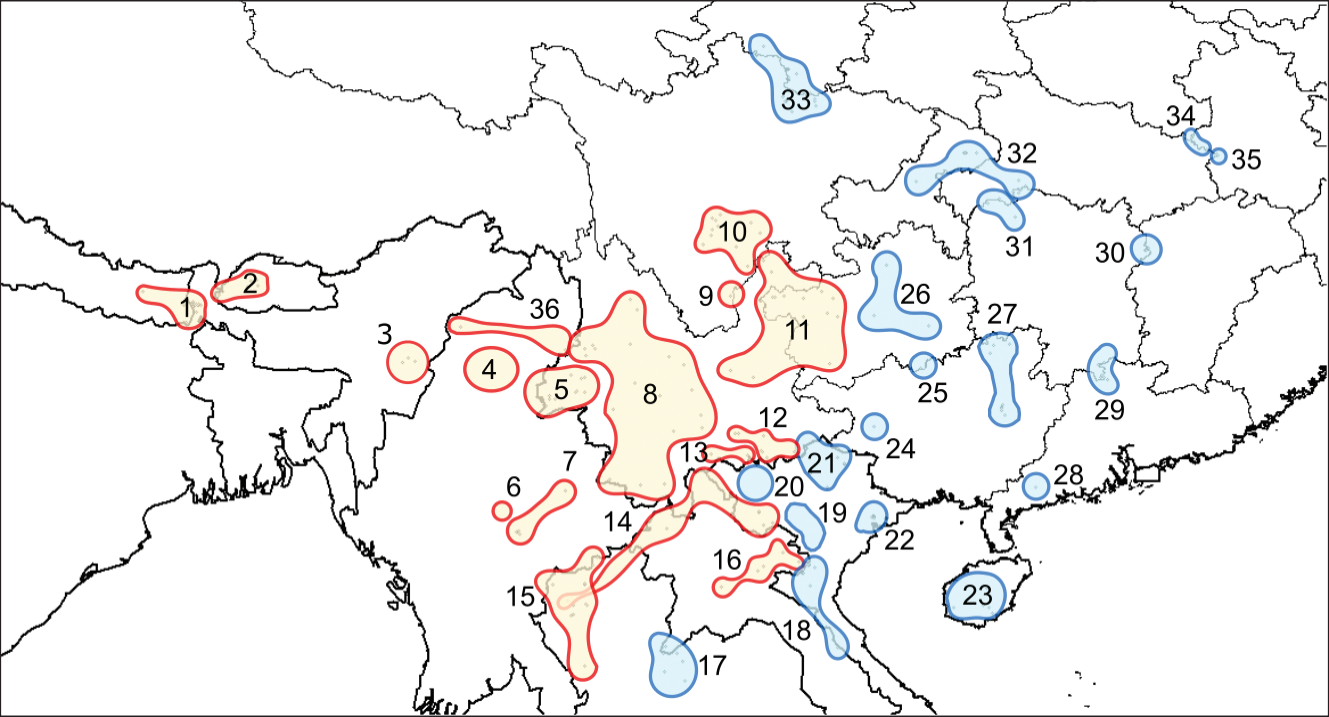
Current distribution map of the genus *Tylototriton*, from South and Central China, to northern Vietnam, Laos, Thailand, Myanmar, India, Bhutan and Nepal (Hernandez 2016; Qian et al. 2017; Grismer et al. 2018; Wang et al. 2018; Grismer et al. 2019; Hernandez et al. 2019; Zaw et al. 2019). In red the distribution areas belonging to the subgenus Tylototriton, and in blue the distribution areas belonging to the sub–genus *Yaotriton* (Dubois and Raffaëlli 2009). The numerical identification corresponds to the different species and undescribed taxa as follow: **1***T. himalayanus***2**T. cf. himalayanus (Bhutan) **3**T. cf. verrucosus (Manipur) **4***T. kachinorum***5***T. verrucosus***6***T. ngarsuensis***7***T. shanorum***8***T. shanjing***9***T. pseudoverrucosus***10***T. taliangensis***11***T. kweichowensis***12***T. yangi***13***T. pulcherrimus***14***T. anguliceps***15***T. uyenoi***16***T. podichthys***17***T. panhai***18***T. notialis***19***Tylototriton* taxon 2, this study **20***Tylototriton* taxon 3, this study **21***T. ziegleri***22***T. vietnamensis***23***T. hainanensis***24**T. cf. hainanensis (Baise) **25**T. cf. wenxianensis (Libo) **26**T. cf. wenxianensis (Guizhou) **27***T. asperrimus***28**T. cf. asperrimus (Guangdong) **29***T. lizhengchangi***30***T. liuyangensis***31***T. broadoridgus***32**T. cf. wenxianensis (Chongquing and Hubei) **33***T. wenxianensis***34***T. dabienicus***35***T. anhuiensis* and **36***T. panwaensis*.

*Tylototriton asperrimus* Unterstein, 1930 was the second salamander species within the genus described after *T. verrucosus*. It was considered a common species due to its relatively wide distribution from central and southern China to northern Vietnam (Bain and Nguyen 2004; Weisrock et al. 2006; van Dijk et al. 2008; Nguyen et al. 2009; Sun et al. 2011; Qin et al. 2012). The increasing amount of field work conducted in these regions, combined with the incorporation of new technologies in taxonomic analyses (e.g., molecular studies, X-ray scans), has since resulted in a vast increase of knowledge on the taxonomy of this genus, turning it into the most speciose genus within the Salamandridae (Fig. 1).

The widely distributed taxon has been revealed to consist of several different species with smaller ranges, and accordingly with a more critical conservation status. For example *T. vietnamensis*, recorded from Bac Giang, Quang Ninh, and Lang Son provinces, Vietnam (Bernardes et al. 2017), currently listed as Endangered (IUCN SSC ASG 2016), *T. ziegleri* from Ha Giang and Cao Bang provinces, Vietnam (Nishikawa et al. 2013b) and Yunnan Province, China (Jiang et al. 2017), listed as Vulnerable (IUCN SSC ASG 2017), *T. broadoridgus*, known from Hunan, China (Shen et al. 2012) and *T. anhuiensis*, known from Anhui, China (Qian et al. 2017), both not yet assessed were some of the species recently described within the *T. asperrimus* species complex.

However, the taxonomic assignments of some populations of *T. asperrimus* have not been completely resolved. The population from Thuong Tien District, Hoa Binh Province, Vietnam, was identified as *T. asperrimus* due to low genetic differences in partial mitochondrial (Yuan et al. 2011; Nishikawa et al. 2013b) and partial nuclear genes (Wang et al. 2018) compared with the Chinese population. Nonetheless, other authors regarded this population as an undescribed species, T. cf. asperrimus, based on genetic differentiation, including complete mitochondrial sequence data (Phimmachak et al. 2015a; Hernandez 2016). Taxonomic decisions, however, should at best not be based on genetic variation alone, but also be accompanied by other evidence, such as morphological and/or ecological differences. Given the high degree of morphological conservatism within the genus *Tylototriton*, identifying phenotypic divergence can be especially challenging (Stuart et al. 2010; Nishikawa et al. 2013b), but nonetheless crucial due to its repercussions on species delimitations.

In order to further understand the taxonomy of species within the *T. asperrimus* complex in Vietnam, we examined specimens of the population from Hoa Binh Province and other newly collected specimens from the region, and compared them with the holotype of *T. asperrimus* from Guangxi, China. We combined molecular and detailed morphological analyses to infer the taxonomic status and phylogenetic relationships among these populations. As a consequence, we herein describe three new taxa of the *T. asperrimus* complex from northern Vietnam.

## Materials and methods

### Sampling

**Figure 2.**
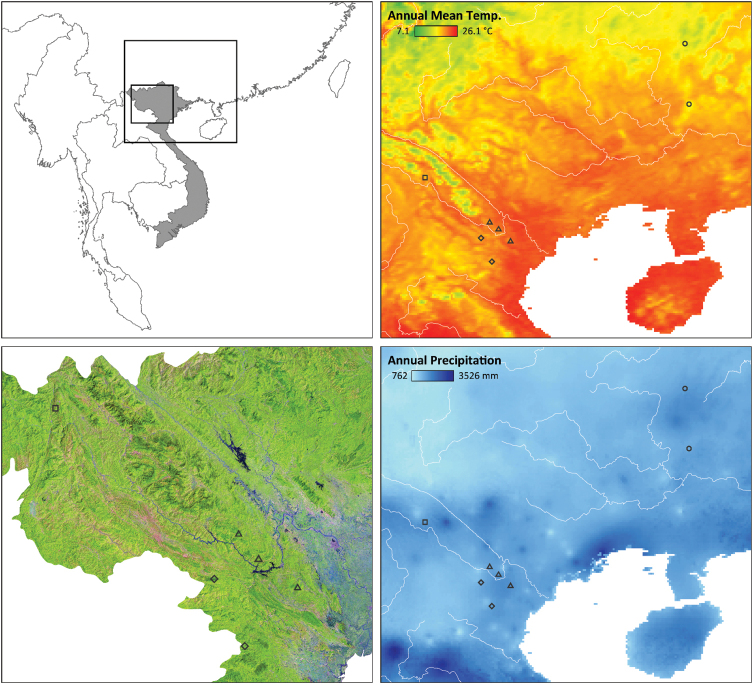
Distribution map of the new populations of *Tylototriton* from North Vietnam, based on the following symbols: **square** (taxon 3, this study) the population from Sin Ho District, Lai Chau Province; **diamond** (taxon 1, this study) the upper one identifies the population from Van Ho District, Son La Province, and the lower one identifies the population from Thuong Xuan District, Thanh Hoa Province; **triangle** (taxon 2, this study) the upper one identifies the population from Tan Son District, Phu Tho Province, the middle one identifies the population from Da Bac District, Hoa Binh Province, and the lower one identifies the population from Lac Son District, Hoa Binh Province. The two populations identified by the **circles** represent *T. asperrimus* sensu stricto from China. High resolution remote sensing land cover information was extracted from “GLAD-UMD and SERVIR-Mekong, Natural annual tree canopy structure and surface water dynamics products, 2017” (lower left panel). Bioclimatic variables (right side) were extracted from remote sensing data provided by Deblauwe et al. (2016).

Field surveys were conducted in northern Vietnam by: 1) A. V. Pham and M. A. Vang in Sa De Phin Commune, Sin Ho District, Lai Chau Province in May 2015, and in Xuan Nha Nature Reserve, Van Ho District, Son La Province on 15 June 2016; 2) H. N. Ngo et al. in Phu Canh Nature Reserve, Da Bac District, Hoa Binh Province on 11 June 2016; 3) T. D. Le et al. in Xuan Son National Park, Du Village, Xuan Son Commune, Tan Son District, Phu Tho Province on 7 July 2016; and 4) T. S. Nguyen in Xuan Lien Nature Reserve, Bat Mot Commune, Thuong Xuan District, Thanh Hoa Province in July 2015 (Fig. 2).

Specimens were anaesthetized and euthanized in a closed vessel with a piece of cotton wool containing ethyl acetate (Simmons 2002), fixed in 80% ethanol for five hours, and subsequently transferred to 70% ethanol for permanent storage. Tissue samples were preserved separately in 70% ethanol prior to fixation. Specimens were subsequently deposited in the collections of the Institute of Ecology and Biological Research (**IEBR**), Hanoi, the Tay Bac University (**TBU**), Son La Province, Vietnam, and the Zoologisches Forschungsmuseum Alexander Koenig (**ZFMK**), Bonn Germany.

### Molecular analyses

Tissue samples from muscle of preserved specimens were extracted using the DNeasy blood and tissue kit, Qiagen (California, USA). A fragment of a mitochondrial gene, the NADH dehydrogenase subunit 2 (ND2), was amplified by PCR mastermix (Fermentas, Burlington, ON, Canada) using the primer pair, Sal_Nd2_F1 (5’- AAGCTTTTGGGCCCATACC-3’) (Nishikawa et al. 2013b) and a newly design primer TyloR1 (5’- GGTCTTTGGTCTYATTATCCTAA -3’). The PCR volume consisted of 21 μl (10 μl of mastermix, 5 μl of water, 2 μl of each primer at 10 pmol/μl and 2 μl of DNA or higher depending on the quantity of DNA in the final extraction solution). The following temperature profile for PCR was used: 95 °C for 5 minutes to activate the taq; with 40 cycles at 95 °C for 30 s, 58 °C for 45 s, 72 °C for 60 s; and the final extension at 72 °C for 6 minutes. PCR products were subjected to electrophoresis through a 1 % agarose gel (UltraPure™, Invitrogen, La Jolla, CA). Gels were stained for 10 min in 1 X TBE buffer with 2 pg/ml ethidium-bromide and visualized under UV light. Successful amplifications were purified to eliminate PCR components using a GeneJET™ PCR Purification kit (Fermentas). Purified PCR products were sent to FirstBase Malaysia for sequencing. We included 12 new samples from five populations distributed in north and north central Vietnam to another 21 known species’ samples of *Tylototriton* (Table 1). Additionally, five species were selected as outgroups: *Echinotriton andersoni*, *E. chinhaiensis*, *Lyciasalamandra atifi*, *Notophthalmus viridescens*, and *Pleurodeles waltl*, to root the tree (Qian et al. 2017; Wang et al. 2018).

**Table 1.** Samples of *Tylototriton* species used in the molecular analyses of this study. Country label key: CH = China; L = Laos; VN = Vietnam.

ID	Species	Voucher	Locality	Genbank no.	Source
1	*T. anhuiensis*	AHU-16-EE-001	Yuexi, Anhui, CH	KY321388	Qian et al. 2017
2	*T. asperrimus* lineage 1	CIB 70063	Longsheng, Guangxi, CH	KC147816	Shen et al. 2012
3	*T. asperrimus* lineage 1	CIB 200807055	Jinxiu, Guangxi, CH	KC147815	Shen et al. 2012
4	*T. asperrimus* lineage 2	CIB XZ20091201	Xinyi, Guangdong, CH	KY800876	Wang et al. 2018
5	*T. broadoridgus*	CIB 200085	Sangzhi, Hunan, CH	KC147814	Shen et al. 2012
6	taxon 1	IEBR 4471	Van Ho, Son La, VN	MT210168	This study
7	taxon 1	IEBR 4473	Van Ho, Son La, VN	MT210169	This study
8	taxon 1	IEBR 4474	Van Ho, Son La, VN	MT210170	This study
9	taxon 1	IEBR 4318	Thuong Xuan, Thanh Hoa, VN	MT210171	This study
10	taxon 1	IEBR 4319	Thuong Xuan, Thanh Hoa, VN	MT210172	This study
11	taxon 2	IEBR 4320	Tan Son, Phu Tho, VN	MT210164	This study
12	taxon 2	IEBR 4321	Tan Son, Phu Tho, VN	MT210165	This study
13	taxon 2	IEBR 4466	Da Bac, Hoa Binh VN	MT201166	This study
14	taxon 2	IEBR 4467	Da Bac, Hoa Binh VN	MT210167	This study
15	taxon 2	VNMN TAO1214 / VFUA.2009.8	Xuan Lien, Lac Son, Hoa Binh, VN	AB769531	Nishikawa et al. 2013b
16	*T. dabienicus* lineage 1	HNNU10042015	Shangcheng, Anhui, CH	KC147811	Nishikawa et al. 2013b
17	*T. dabienicus* lineage 2	CIB 08042905-2	Yuexi, Anhui, CH	KY800853	Wang et al. 2018
18	*T. hainanensis*	CIB 20081048	Diaoluoshan, Hainan, CH	KC147817	Nishikawa et al. 2013b
19	*T. liuyangensis*	CSUFT20100108	Liuyang, Hunan, CH	KJ205598	Yang et al. 2014
20	*T. lizhengchangi*	KUHE 42317	Yizhang, Hunan, CH	AB769533	Nishikawa et al. 2013b
21	*T. notialis*	VNMN TAO1235	Pu Hoat, Nghe An, VN	AB769536	Nishikawa et al. 2013b
22	*T. panhai*	NUOL 00437	Botene, Xaignabouli, L	KT304306	Phimmachak et al. 2015a
23	taxon 3	IEBR 4477	Sin Ho, Lai Chau, VN	MT210167	This study
24	taxon 3	IEBR 4476	Sin Ho, Lai Chau, VN	MT210162	This study
25	*T. taliangensis*	KUHE 43361	Pet Trade	AB769543	Nishikawa et al. 2013b
26	*T. verrucosus*	KIZ 201306058	Husa, Yunnan, CH	AB922820	Nishikawa et al. 2014
27	*T. vietnamensis*	KUHE 55172	Yen Tu, Bac Giang, VN	AB769538	Nishikawa et al. 2013b
28	*T. vietnamensis*	IEBR A.2014.43	Hoanh Bo, Quang Ninh, VN	KX609961	Bernardes et al. 2017
29	*T. vietnamensis*	IEBR A.2014.45	Loc Binh, Lang Son, VN	KX609963	Bernardes et al. 2017
30	*T. wenxianensis* lineage 1	CIB 20090527	Wenxian, Gansu, CH	KC147813	Nishikawa et al. 2013b
31	*T. wenxianensis* lineage 2	CIB Wg20090730001	Libo, Guizhou, CH	KY800842	Wang et al. 2018
32	*T. wenxianensis* lineage 3	CIB WH10003	Wufeng, Hubei, CH	KY800865	Wang et al. 2018
33	*T. ziegleri*	VNMN 3390	Quan Ba, Ha Giang, VN	AB769539	Nishikawa et al. 2013b

The sequences were aligned in Clustal X v2 (Thompson et al. 1997) with default settings. Data were analyzed using maximum parsimony (MP) and maximum likelihood (ML) as implemented in PAUP 4.0b10 (Swofford 2001), and Bayesian analysis in MrBayes 3.2 (Ronquist et al. 2012). For MP analysis, heuristic analysis was conducted with 100 random taxon addition replicates using tree-bisection and reconnection (TBR) branch swapping algorithm, with no upper limit set for the maximum number of trees saved. Bootstrap support (BP) (Felsenstein 1985) was calculated using 1,000 pseudo-replicates and 100 random taxon addition replicates. All characters were equally weighted and unordered. For ML analysis, we used the optimal evolution model as selected by ModelTest v3.7 (Posada and Crandall 1998). To estimate BP in the ML analysis, a simple taxon addition option and 100 pseudo-replicates were employed. We considered BP values of ≥ 70 % to represent strong support (Hillis and Bull 1993).

For Bayesian analyses, we used the optimal model, GTR+I+G as selected by Modeltest v3.7, for ML and combined Bayesian analyses. Two simultaneous analyses with four Markov chains (one cold and three heated) were run for 10 million generations with a random starting tree and sampled every 1,000 generations. Log-likelihood scores of sample points were plotted against generation time to determine stationarity of Markov chains. The cutoff point for the burn-in function was set to 21, equivalent to 21,000 generations, in the Bayesian analysis, as -lnL scores reached stationarity after 21,000 generations in both runs. Nodal support was evaluated using Bootstrap in PAUP and posterior probability in MrBayes v3.2. Uncorrected pairwise divergences were calculated in PAUP*4.0b10.

We selected the relaxed-clock method (Drummond et al. 2006) to estimate divergence times. The obtained dataset was used as input for the computer program BEAST v1.8.0 (Drummond and Rambaut 2007). *A priori* criteria for the analysis were set in the program BEAUti v1.8.0. One calibration point, the split between the clade containing *Tylototriton vietnamensis* + *T. panhai* and the clade consisting of *T. asperrimus* and other related species, estimated for 12.4 ± 2.3 million years ago (MYA) (Wang et al. 2018), was used to calibrate the phylogeny. A general time-reversible (GTR) model using gamma + invariant sites with four gamma categories was employed along with the assumption of a relaxed molecular clock. As for the priors, we used all default settings, except for the Tree Prior category that was set to Yule Process, as recommended for species-level analyses. The codon-partitioned dataset was used for a single run. In addition, a random tree was employed as a starting tree. The length chain was set to 10^7^, and the Markov chain was sampled every 1,000 generations. After the dataset with the above settings was analyzed in BEAST, the resulting likelihood profile was then examined by the program Tracer v1.6 to determine the burn-in cutoff point. The final tree with calibration estimates was computed using the program TreeAnnotator v1.8.0 as recommended in the BEAST program manual.

### Morphological examination

**Figure 3.**
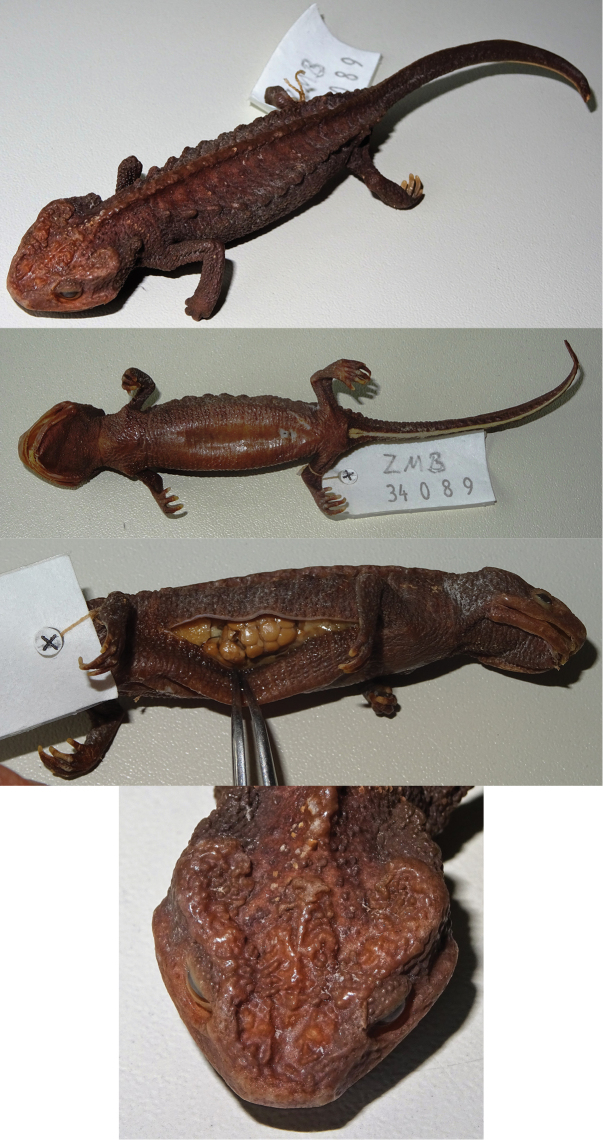
Holotype of *Tylototriton asperrimus* (ZMB 34089). In sequence: dorsal view; ventral view; lateral view with detail of ovaries; and detail of dorsal view of the head. Photographs T. Ziegler.

All specimens were sexed by evaluating the size of the opening of the cloacal fissure: females show a puncture-like opening and males a wider slit-like opening. The holotype of *T. asperrimus* (ZMB 34089), collected from Guangxi Province, China, was loaned from the Zoologisches Museum Berlin (Museum für Naturkunde Berlin) and evaluated as a female (Fig. 3). In addition we investigated two other Vietnamese female specimens, one from IEBR: JJLR01195 from Pu Hoat Nature Reserve, Nghe An Province (*T. notialis*) and another from the Vietnam Forestry University (VFU) in Hanoi: VFUA.2009.8 (also known as voucher Tao1214 in Nishikawa et al. [2013b]) from Thuong Tien Nature Reserve, Hoa Binh Province (T. cf. asperrimus). Morphological comparisons were only performed among animals of the same sex, and only males had a sufficiently large number of specimens (*N*) to perform statistical analysis.

A total of 23 morphological characters were measured following Bernardes et al. (2017) to the nearest 0.01 mm with a digital caliper as follows: snout-vent length (**SVL**); head length (**HL**); head width (**HW**) measured behind the eyes and before the beginning of the parotoids; maximum head width (**MHW**); parotoid width (**PW**); maximum parotoid height (**PH**); eye length (**EL**); inter-eye distance (**IE**); inter-narial distance (**IN**); eye-narial distance (**EN**); lower jaw length (**LJL**) from tip of lower jaw to jaw angle; maximum upper eyelid length (**UEL**); humerus length (**HUM**); radius length (**RAD**); femur length (**FEM**); tibia length (**TIB**); axilla to groin (**AG**); trunk length (**TkL**) from wrinkle of throat to anterior tip of vent; length of the 5^th^ anterior dorsal nodule (**L5N**); width of vertebral cord (**WVr**) measured at the height of the 5^th^ nodule; cloaca length (**ClL**) length of cloaca muscle; tail length (**TL**); tail height (**TH**). The following ratios were calculated based on the measures above: total forelimb length (**FORE**); total hindlimb length (**HIND**); hind-limb to forelimb lengths (**HIND/FORE**); the relative length of radius to humerus (**RAD/HUM**); tibia to femur (**TIB/FEM**); and tail length to tail height (**TL/TH**).

The morphological comparison between the new taxa and their congeners were based on the specimen examination and the following literature: Fei et al. (1984), Böhme et al. (2005), Stuart et al. (2010), Shen et al. (2012), Nishikawa et al. (2013a), Nishikawa et al. (2013b), and Yang et al. (2014). When measurements were involved, only the ones taken in similar ways were found suitable for comparison and used as reference.

### Statistical analysis

We first compared the morphological characters of males between the two clades originating on both sides of the Da River: the western clade from Son La and Thanh Hoa provinces (referred to as taxon 1) and the eastern clade from Hoa Binh and Phu Tho provinces (referred to as taxon 2; for reference see Fig. 2). Subsequently we compared the above-mentioned males (jointly referred to as T. cf. asperrimus) and the males originating from Lai Chau Province (referred to as taxon 3).

The statistical analyses had to be conducted on different subsets of morphological characters according to data availability. Morphological characters that could not be obtained for all the species had to be excluded from the overall analysis. These included: PW, PH, EL, IE, UEL, AG, and ClL. Whether the measured morphological characters showed a linear increase with body size was analyzed through correlation analyses (see Suppl. material 1). Accordingly, measurements of morphological characters and character ratios were standardized by SVL (R[character]: % SVL) to exclude the effect of body size, and log-transformed. A Principal Component Analysis (PCA) was tested by a one-way Analysis of Variance (ANOVA) between populations. Because morphological traits within individuals are not independent of each other, comparisons between different morphological traits of species were based on Multivariate Analysis of Variance (MANOVA) and proceeding to ANOVA and Tukey HSD test only if the MANOVA yielded a significant result (i.e., ‘protected ANOVA’ (van Ende 2001). Roy’s Greatest Root was chosen as test of significant differences among groups in the MANOVA procedure.

Significance levels were set to 95 %. All statistical analyses were performed in R v 3.1.2, the vegan package was used to calculate PCA (Oksanen et al. 2015).

### Macroclimatic information

Climatic information at the sample sites were extracted from remote sensing data (Deblauwe et al. 2016). Representing averages across last decades with a spatial resolution of 0.1°, the following bioclimatic variables were available: Annual Mean Temperature BIO1, Mean Diurnal Range BIO2, Isothermality BIO3, Temperature Seasonality BIO4, Max Temperature of Warmest Month BIO5, Min Temperature of Coldest Month BIO6, Temperature Annual Range BIO7, Mean Temperature of Wettest Quarter BIO8, Mean Temperature of Driest Quarter BIO9, Mean Temperature of Warmest Quarter BIO10, Mean Temperature of Coldest Quarter BIO11, Annual Precipitation BIO12, Precipitation of Wettest Month BIO13, Precipitation of Driest Month BIO14, Precipitation Seasonality BIO15, Precipitation of Wettest Quarter BIO16, Precipitation of Driest Quarter BIO17, Precipitation of Warmest Quarter BIO18, and Precipitation of Coldest Quarter BIO19.

## Results

### Molecular analyses

**Figure 4.**
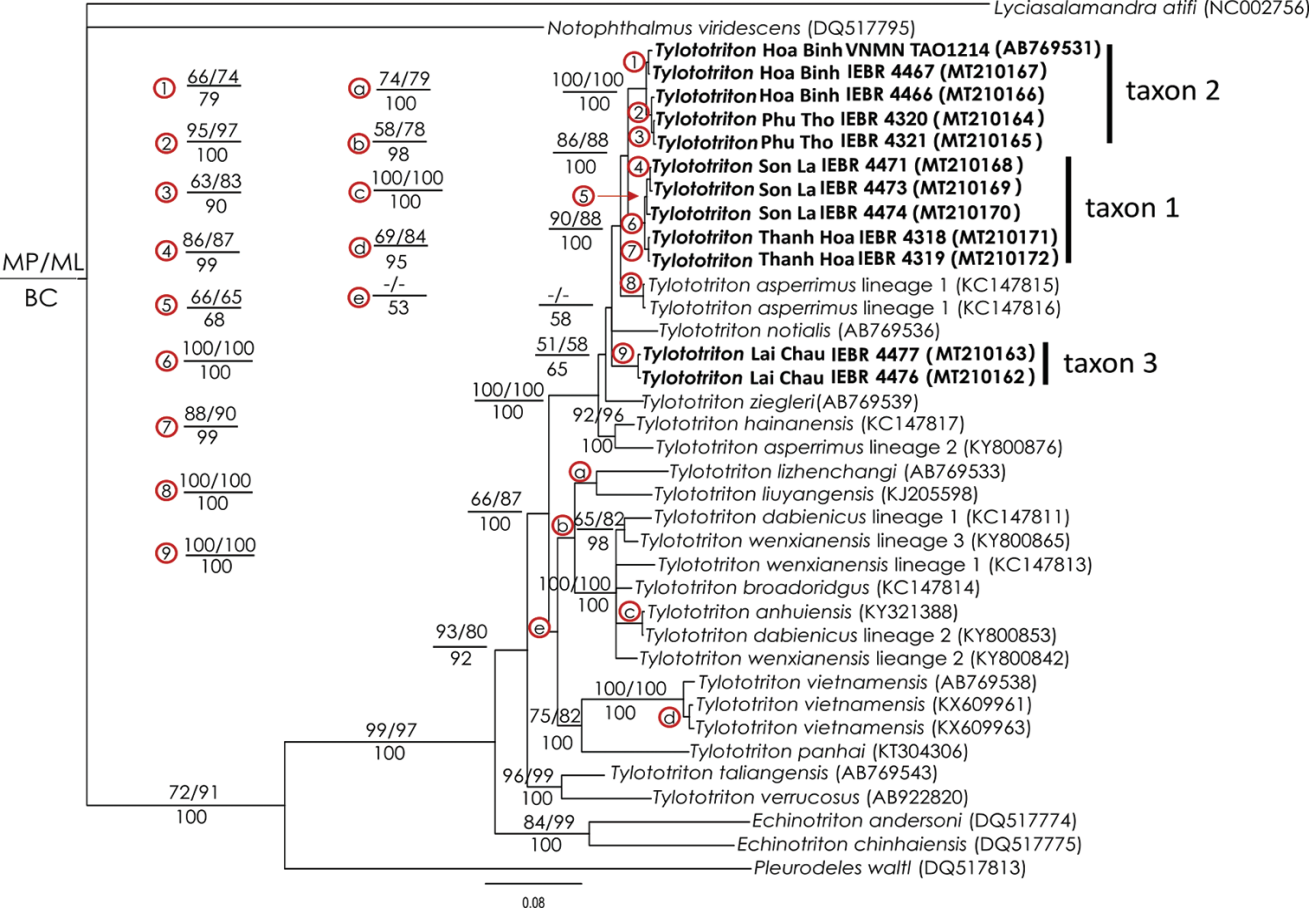
Phylogram based on the Bayesian analysis. Number above and below branches are MP/ML bootstrap values and Bayesian posterior probabilities (> 50 %), respectively. Dashes represent values < 50 %. Sample AB769531 is from Nishikawa et al. 2013b.

The combined matrix contained 1036 aligned characters. Of those, 370 were parsimony informative. MP analysis of the dataset recovered 2 most parsimonious trees with 1400 steps (CI = 0.54; RI = 0.65). Our phylogenetic analyses recovered the Vietnamese T. cf. asperrimus as a sister taxon to *T. asperrimus* from China with strong support values from all analyses (MP_BP_ = 90, ML_BP_ = 88, PP = 100) (Fig. 4). The genetic differences between Vietnamese populations and the Chinese lineage were 3.3 to 3.6 % for the population from Son La Province; 3.2 to 3.4 % for the population from Thanh Hoa Province; 3.3 to 3.6 % for the population from Phu Tho Province; 3.2 to 3.6 % for the population from Da Bac District, Hoa Binh Province; and 3.4 to 3.5 % for the population from Lac Son District, Hoa Binh Province, respectively (Table 2).

**Table 2.** Uncorrected p-distances of the mitochondrial DNA sequences used in this study for members of the *Tylototriton asperrimus* species complex.

ID	Taxon–Locality	1	2	3	4	5	6	7	8	9	10	11	12	13	14
1	*T. asperrimus*–China	–													
2	*T. asperrimus*–China	0.001	–												
3	taxon 1–Son La	0.034	0.034	–											
4	taxon 1–Son La	0.036	0.035	0.001	–										
5	taxon 1–Thanh Hoa	0.034	0.033	0.005	0.006	–									
6	taxon 1–Son La	0.034	0.033	0.004	0.005	0.005	–								
7	taxon 1–Thanh Hoa	0.033	0.032	0.005	0.006	0.000	0.005	–							
8	taxon 2–Phu Tho	0.034	0.033	0.029	0.030	0.028	0.029	0.028	–						
9	taxon 2–Phu Tho	0.036	0.035	0.030	0.031	0.029	0.030	0.029	0.001	–					
10	taxon 2–Hoa Binh	0.036	0.034	0.030	0.030	0.028	0.029	0.028	0.002	0.003	–				
11	taxon 2–Hoa Binh	0.033	0.032	0.026	0.027	0.025	0.026	0.025	0.005	0.006	0.005	–			
12	taxon 2–Hoa Binh	0.035	0.034	0.029	0.031	0.029	0.029	0.028	0.007	0.008	0.007	0.002	–		
13	taxon 3–Lai Chau	0.042	0.041	0.039	0.040	0.038	0.039	0.038	0.044	0.045	0.044	0.041	0.044	–	
14	taxon 3–Lai Chau	0.041	0.041	0.039	0.040	0.036	0.039	0.036	0.044	0.045	0.044	0.041	0.044	0.002	–

Furthermore, our genetic analyses identified different lineages within the Vietnamese clade of T. cf. asperrimus. The genetic variation between taxon 1 and taxon 2 varied between 2.5 % (between Thanh Hoa and Hoa Binh populations) and 3.1 % (between Son La and Phu Tho populations). In contrast, within-population differences were only 0.0 to 0.6 % in taxon 1 and 0.1 to 0.9 % in taxon 2.

The population from Lai Chau Province turned out to be a distinct and basal lineage within a weakly supported clade, including *T. notialis*, *T. asperrimus* from China, and taxon 1 and taxon 2 from Vietnam (Fig. 4). In this case the genetic differences of taxon 3 to the topotypical population of *T. asperrimus* ranged between 4.1 to 4.2 % to taxon 1 between 3.6 to 4.0 %, and to taxon 2 between 4.1 to 4.5 % (see Table 2 for genetic distances). Our time estimates are very similar to those generated by Wang et al. (2018), and the results show that *T. asperrimus* from China split from taxon 1 about 2.5 MYA (95% highest posterior densities – 95% HPD = 1.4–3.7), while taxon 3 diverged from the two taxa approximately 3.4 MYA (95% HPD = 2.3–4.8) (see Suppl. material 2).

### Morphological examination

Vietnamese species compared to the Chinese holotype

This comparison was only based on three female specimens: the holotype of *T. asperrimus*, one from Hoa Binh Province (taxon 2), and one from Nghe An Province (*T. notialis*) (Table 3). Due to the lack of replicates it was not possible to perform statistical analyses between the Chinese and the Vietnamese clades. After correcting the absolute measures to ratios of snout-vent length, the most prominent differences between the female of taxon 2 and the female holotype of *T. asperrimus* from China were: a wider and longer head (MHW = 28.99, HL = 29.15 in taxon 2 vs. MHW = 25.35, HL = 26.60 in *T. asperrimus*), a longer lower jaw (LJL = 17.40 in taxon 2 vs. 14.85 in *T. asperrimus*), and higher values for most of the measured head features (including the distance between the eyes) for taxon 2. The exceptions were found in the distance between eye and nostril (EN = 6.37 in *T. asperrimus* vs. 4.86 in taxon 2) and head width (HW = 18.54 in *T. asperrimus* vs. 13.67 in taxon 2) which in these cases the values were higher in *T. asperrimus*. The female from taxon 2 also had higher values for tail length (TL = 85.62 in taxon 2 vs. 77.28 in *T. asperrimus*), cloacal muscles (ClL = 11.39, ClW = 7.63 in taxon 2 vs. ClL = 7.01, ClW = 3.65 in *T. asperrimus*), and vertebra width (WVr = 4.46 in taxon 2 vs. 2.80 in *T. asperrimus*). The female from China had a longer trunk length (TkL = 74.58 in *T. asperrimus* vs. 69.67 in taxon 2). The female from Nghe An Province differed by having the smallest eye length, the shortest distance between both eyes, the smallest glandular warts and by having the longest limbs, while other measurements did not separate it from other lineages.

**Table 3.** Morphological comparisons between the available females. Measures as absolute values (in mm) and ratios of characters to snout vent length (% SVL) between *Tylototriton asperrimus* holotype from China (ZMB 34089), T. cf. asperrimus from Thuong Tien Nature Reserve, Hoa Binh Province, Vietnam (taxon 2) (VFUA.2009.8), and *T. notialis* (JJLR01195) from Pu Hoat Nature Reserve, Nghe An Province, Vietnam. For abbreviations see Materials and methods.

Character	Absolute measures			Ratios to SVL		
	*T. asperrimus*	taxon 2	*T. notialis*	*T. asperrimus*	taxon 2	*T. notialis*
**SVL**	73.45	76.82	76.16	–	–	–
**MHW**	18.62	22.27	19.64	25.35	28.99	25.79
**HW**	13.62	10.50	–	18.54	13.67	–
**HL**	19.54	22.39	20.86	26.60	29.15	27.39
**PL**	10.74	13.03	12.78	14.62	16.96	16.78
**PH**	5.78	7.45	7.54	7.87	9.70	9.90
**EL**	3.17	3.86	2.94	4.32	5.02	3.86
**EN**	4.68	3.73	3.79	6.37	4.86	4.98
**IN**	5.78	7.37	6.46	7.87	9.59	8.48
**IE**	9.40	10.13	9.11	12.80	13.19	11.96
**LJL**	10.91	13.37	–	14.85	17.40	–
**UEL**	4.27	5.06	–	5.81	6.59	–
**HUM**	8.98	7.91	8.63	12.23	10.30	11.33
**RAD**	16.04	16.88	18.11	21.84	21.97	23.78
**FEM**	7.17	7.69	9.89	9.76	10.01	12.99
**TIB**	17.07	18.29	18.37	23.24	23.81	24.12
**FORE**	25.02	24.79	26.74	34.06	32.27	35.11
**HIND**	24.24	25.98	28.26	33.00	33.82	37.11
**HIND.FORE**	0.97	1.05	1.06	1.32	1.36	1.39
**RAD.HUM**	1.79	2.13	2.10	2.43	2.78	2.76
**TIB.FEM**	2.38	2.38	1.86	3.24	3.10	2.44
**TL**	56.76	65.77	65.14	77.28	85.62	85.53
**TH**	7.87	9.44	9.39	10.71	12.29	12.33
**TL.TH**	7.21	6.97	6.94	9.82	9.07	9.11
**ClL**	5.15	8.75	5.87	7.01	11.39	7.71
**ClW**	2.68	5.86	5.25	3.65	7.63	6.89
**WVr**	2.06	3.43	2.49	2.80	4.46	3.27
**L5W**	2.39	2.66	2.29	3.25	3.46	3.01
**AG**	37.12	37.56	–	50.54	48.89	–
**TkL**	54.78	53.52	–	74.58	69.67	–

Comparisons within T. cf. asperrimus from Vietnam

The comparison between taxon 1 and taxon 2 included only males. Absolute measures and ratios of species’ morphological traits corrected by snout vent length are shown in Table 4. Taxon 1 and taxon 2 did not differ in their respective SVL (*t*-test = -1.55, DF = 18, *p* = 0.14). Taxon 1 presented wider head than taxon 2 (MHW = 27.37 ± 1.67, HW = 19.86 ± 0.95 for taxon 1 vs. MHW = 25.11 ± 0.81, HW = 18.75 ± 0.99 taxon 2). The ratios of EN and IN also differed between lineages, with taxon 1 having a relatively longer snout (EN = 6.16 ± 0.68) than taxon 2 (5.50 ± 0.37) and taxon 2 having a wider snout (IN = 8.58 ± 0.57) than taxon 1 (7.94 ± 0.85). Taxon 2 showed the highest variation range in limb data. The ratio of FEM was longer in taxon 2 (12.59 ± 0.78) than in taxon 1 (10.99 ± 0.67), as well as the ratio of TIB (23.69 ± 2.35 in taxon 2 vs. 22.41 ± 0.61 in taxon 1), which together also resulted in longer hind limbs (HIND = 36.28 ± 2.89 in taxon 2 vs. 33.40 ± 0.99 in taxon 1). The ratio of the fore-limbs on the other hand was alike between lineages. Although taxon 2 presented lower minimum values for both RAD (18.80) and HUM (9.30) than in taxon 1 (RAD = 20.08 and HUM = 11.04). The tail in taxon 1 was longer (87.06 ± 5.25) and less high (11.79 ± 1.14) than in taxon 2 (TL = 84.61 ± 5.20, TH = 12.43 ± 2.46). Taxon 1 showed longer trunk (70.28 ± 3.41) than taxon 2 (68.78 ± 1.24).

**Table 4.** Morphological measurements between the *Tylototriton* males from Son La and Thanh Hoa provinces (taxon 1), from Phu Tho and Hoa Binh provinces (taxon 2), and from Lai Chau Province (taxon 3). Measures as absolute values (in mm) and ratios of characters to snout vent length (% SVL). Values are presented as mean ± standard deviation above minimum and maximum ranges, and for abbreviations see Materials and methods.

Character	*N*			Absolute measures			Ratios to SVL		
				taxon 1	taxon 2	taxon 3	taxon 1	taxon 2	taxon 3
**SVL**	10	11	5	64.98 ± 1.87	66.59 ± 2.81	65.14 ± 2.12			
				62.00 –67.95	61.57 –70.69	63.20–68.71			
**MHW**	10	11	5	17.78 ± 1.11	16.71 ± 0.64	17.12 ± 0.43	27.37 ± 1.67	25.11 ± 0.81	26.30 ± 0.86
				16.00 –19.63	15.66 –17.90	16.68 –17.60	24.71 –29.91	24.11 –26.31	25.61 –27.75
**HW**	10	11	3	12.89 ± 0.48	12.47 ± 0.63	10.00 ± 0.53	19.86 ± 0.95	18.75 ± 0.99	15.29 ± 1.04
				12.10 –13.70	10.98 –13.13	9.56 –10.59	18.44 –21.29	16.98 –20.45	14.34 –16.40
**HL**	10	11	5	18.67 ± 0.71	18.79 ± 0.89	18.71 ± 0.93	28.75 ± 1.16	28.23 ± 1.23	28.72 ± 0.75
				17.24 –19.54	17.64 –20.39	17.42 –19.95	26.63 –30.32	25.41 –29.80	27.56 –29.62
**PL**	10	5	5	9.74 ± 0.80	9.64 ± 0.58	10.34 ± 0.43	14.99 ± 1.31	14.80 ± 0.46	15.88 ± 0.79
				8.52 –10.92	8.79 –10.27	9.92 –11.02	13.02 –16.64	14.28 –15.48	14.82 –16.92
**PH**	10	5	5	5.70 ± 0.68	5.28 ± 0.73	5.77 ± 0.21	8.78 ± 1.10	8.10 ± 0.97	8.87 ± 0.18
				4.77 –6.98	4.50 –6.11	5.58 –6.12	7.13 –10.81	7.01 –9.52	8.64 –9.15
**EL**	10	5	5	3.14 ± 0.24	3.24 ± 0.12	3.26 ± 0.23	4.84 ± 0.41	4.99 ± 0.32	5.01 ± 0.31
				2.60 –3.40	3.06 –3.40	3.02 –3.49	3.89 –5.24	4.54 –5.31	4.68 –5.45
**EN**	10	11	5	4.00 ± 0.41	3.66 ± 0.25	4.40 ± 0.46	6.16 ± 0.68	5.50 ± 0.37	6.77 ± 0.78
				3.43 –4.75	3.24 –4.11	3.74 –5.05	5.23 –7.19	4.75 –6.03	5.74 –7.88
**IN**	10	11	5	5.16 ± 0.60	5.71 ± 0.40	5.67 ± 0.54	7.94 ± 0.85	8.58 ± 0.57	8.70 ± 0.63
				4.03 –5.97	5.17 –6.43	5.01 –6.26	6.13 –9.18	7.79 –9.88	7.93 –9.35
**IE**	10	5	5	8.56 ± 0.17	8.61 ± 0.48	8.78 ± 0.56	13.18 ± 0.37	13.22 ± 0.45	13.48 ± 0.73
				8.24 –8.82	8.11 –9.17	8.00 –9.50	12.53 –13.67	12.64 –13.85	12.66 –14.58
**LJL**	10	11	3	11.20 ± 0.61	12.59 ± 1.76	10.66 ± 0.62	17.25 ± 1.05	18.88 ± 2.30	16.29 ± 0.98
				10.20 –12.11	10.05 –14.73	10.00 –11.24	15.54 –18.88	14.22 –21.33	15.63 –17.41
**UEL**	10	5	3	4.59 ± 0.36	4.42 ± 0.33	4.80 ± 0.15	7.07 ± 0.65	6.79 ± 0.64	7.35 ± 0.49
				4.00 –5.07	3.92 –4.83	4.70 –4.97	5.98 –7.95	6.11 –7.42	6.90–7.86
**HUM**	10	11	5	7.86 ± 0.40	7.98 ± 0.99	9.03 ± 0.98	12.11 ± 0.74	11.96 ± 1.15	13.89 ± 1.69
				7.15 –8.61	5.97 –9.22	7.81 –10.47	11.04 –13.89	9.30 –13.54	11.99–16.57
**RAD**	10	11	5	14.01 ± 0.61	14.46 ± 1.13	14.20 ± 1.12	21.56 ± 0.72	21.72 ± 1.52	21.78 ± 1.21
				13.21 –14.96	12.50 –15.96	12.71 –15.86	20.08 –22.42	18.80 –23.60	19.84–23.08
**FEM**	10	11	4	7.14 ± 0.39	8.40 ± 0.81	8.17 ± 0.79	10.99 ± 0.67	12.59 ± 0.78	12.44 ± 0.99
				6.31 –7.54	7.08 –9.59	7.46 –8.93	9.67 –11.83	11.50 –13.97	11.65–13.71
**TIB**	10	11	4	14.57 ± 0.60	15.78 ± 1.77	15.29 ± 0.82	22.41 ± 0.61	23.69 ± 2.35	23.30 ± 0.86
				13.73 –15.75	13.14 –17.91	14.25 –16.03	21.27 –23.19	18.59 –26.67	22.24–24.34
**FORE**	10	11	5	21.87 ± 0.62	22.44 ± 1.86	23.23 ± 1.54	33.67 ± 0.89	33.68 ± 2.11	35.67 ± 2.13
				21.11 –23.08	19.48 –25.18	21.75 –25.13	32.43 –35.35	30.36 –36.97	33.88–38.94
**HIND**	10	11	4	21.70 ± 0.78	24.18 ± 2.41	23.46 ± 1.58	33.40 ± 0.99	36.28 ± 2.89	35.74 ± 1.78
				20.34 –22.80	21.14 –27.06	21.71 –24.80	31.51 –34.96	30.98 –39.90	33.89–38.05
**HIND/FORE**	10	11	4	0.99 ± 0.03	1.08 ± 0.05	1.03 ± 0.06	1.53 ± 0.06	1.62 ± 0.09	1.57 ± 0.12
				0.95 –1.03	0.99 –1.15	0.99 –1.12	1.44 –1.65	1.40 –1.71	1.44–1.72
**RAD/HUM**	10	11	5	1.79 ± 0.13	1.34 ± 0.54	1.59 ± 0.20	2.75 ± 0.20	2.03 ± 0.89	2.43 ± 0.27
				1.55 –2.02	0.86 –2.26	1.35 –1.83	2.47 –3.12	1.26 –3.53	2.14–2.80
**TIB/FEM**	10	11	4	2.05 ± 0.13	1.88 ± 0.15	1.88 ± 0.10	3.15 ± 0.18	2.83 ± 0.30	2.87 ± 0.21
				1.91 –2.32	1.50 –2.09	1.78 –2.00	2.90 –3.56	2.12 –3.40	2.66–3.10
**TL**	10	11	4	54.94 ± 3.02	57.96 ± 4.12	55.97 ± 2.81	84.61 ± 5.20	87.06 ± 5.25	85.92 ± 2.47
				50.47 –60.71	53.64 –64.13	53.16 –59.70	77.37 –93.13	76.88 –94.16	82.34–88.00
**TH**	9	10	5	8.06 ± 1.67	7.87 ± 0.71	8.00 ± 0.64	12.43 ± 2.46	11.79 ± 1.14	12.28 ± 0.90
				6.13 –11.34	6.69 –8.83	7.03 –8.57	9.47 –17.28	9.82 –13.76	10.97–13.10
**TL/TH**	9	10	4	7.06 ± 1.35	7.49 ± 1.04	6.97 ± 0.75	10.93 ± 2.22	11.22 ± 1.51	10.72 ± 1.23
				4.80 –8.82	6.21 –9.59	6.31 –8.02	7.31 –13.62	8.79 –14.07	9.78–12.52
**ClL**	10	5	4	8.98 ± 0.96	9.41 ± 1.70	8.34 ± 0.93	13.82 ± 1.43	14.39 ± 1.91	12.78 ± 0.98
				8.02 –10.96	8.13 –12.12	7.54 –9.66	12.39 –16.98	12.67 –17.15	11.68–14.06
**ClW**	10	5	0	5.06 ± 0.67	4.63 ± 0.66		7.79 ± 1.03	7.08 ± 0.61	
				4.16 –5.91	4.09 –5.77		6.38 –9.06	6.64 –8.16	
**WVr**	10	10	5	1.99 ± 0.23	1.93 ± 0.27	2.30 ± 0.17	3.06 ± 0.37	2.90 ± 0.37	3.53 ± 0.25
				1.58 –2.30	1.43 –2.37	2.10 –2.52	2.44 –3.53	2.23 –3.43	3.32–3.90
**L5N**	10	11	5	1.73 ± 0.26	1.93 ± 0.24	2.17 ± 0.63	2.67 ± 0.39	2.91 ± 0.41	3.31 ± 0.87
				1.41 –2.12	1.39 –2.31	1.44 –3.04	2.12 –3.21	2.02 –3.60	2.25–4.42
**AG**	10	5	5	30.28 ± 2.41	30.26 ± 4.05	30.56 ± 1.80	46.59 ± 3.42	46.31 ± 3.91	46.92 ± 2.47
				26.26 –35.45	27.36 –37.20	27.97 –32.66	40.68 –54.01	42.64 –52.62	44.26–50.13
**TkL**	10	10	3	45.66 ± 2.43	45.81 ± 2.16	43.31 ± 1.58	70.28 ± 3.41	68.78 ± 1.24	66.22 ± 3.86
				41.48 –50.25	42.19 –50.20	42.10 –45.10	64.26 –76.57	67.35 –71.01	62.17–69.86

**Figure 5.**
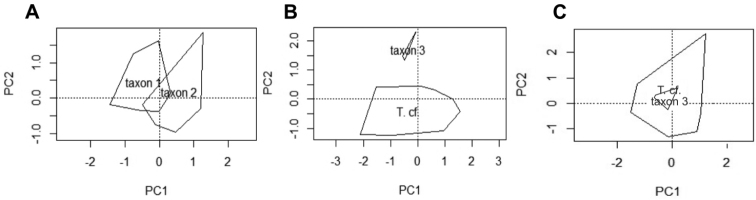
Scatterplot between PC1 and PC2 of the morphological characters corrected to SVL and log–transformed, for **A** taxon 1 and taxon 2 of the Vietnamese Tylototriton cf. asperrimus**B** the head- and dorso- related data of taxon 3 from Lai Chau Province and T. cf. asperrimus from Vietnam *sensu lato*; and **C** the limb related data of taxon 3 from Lai Chau Province and T. cf. asperrimus from Vietnam *sensu lato.* In the graphics *T.* cf. refers to T. cf. asperrimus.

The statistical analysis was based on nine males of taxon 1 and ten males of taxon 2. A PCA analysis resulted in six principal components (PC) explaining 87 % of the total variation. The first two PCs accounted for 52 % of the variation. The scatterplot between PC1 and PC2 showed a clear separation of the two clades, with only a small overlap area (Fig. 5A).

**Figure 6.**
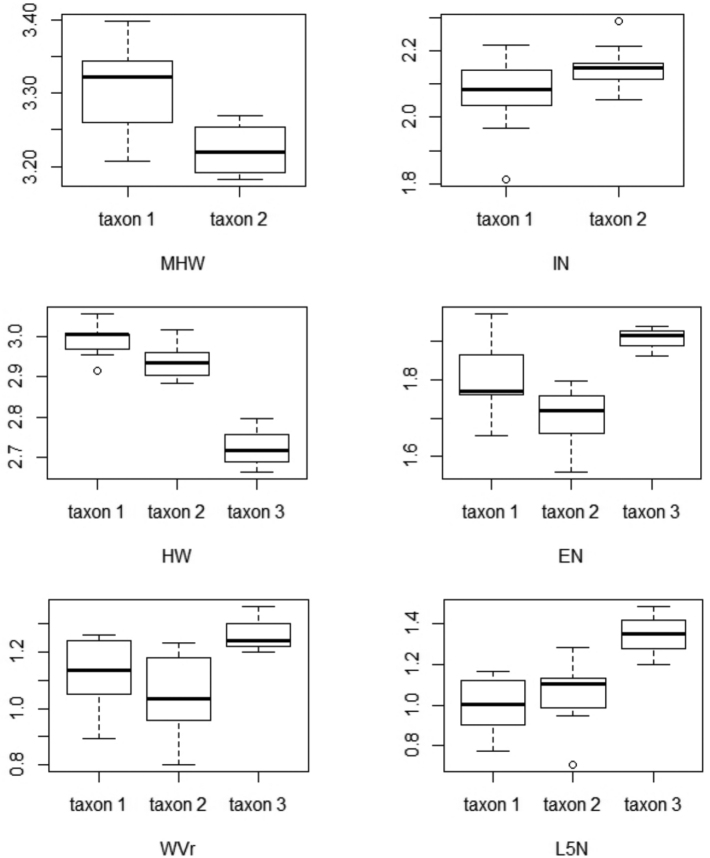
Boxplot of the most differing characters related to head and dorsal values between taxon 1, taxon 2, and taxon 3. Characters were corrected to SVL and log–transformed. For abbreviations see Materials and methods.

The head related data (MANOVA: *F*_1, 17_ = 11.75, DF = 6, *p* < 0.001), and the limb related data (MANOVA: *F*_1, 17_ = 5.10, DF = 9, *p* = 0.01) were significantly different between the two lineages. Tail and dorsal morphological traits were not significantly different (MANOVA: *F*_1, 17_ = 1.42, DF = 6, *p* = 0.3). Our results identified MHW, HW, EN, IN, RAD/HUM, FEM, TIB/FEM, HIND, and HIND/FORE as important traits separating both lineages (Table 5). Taxon 1 has a wider head (both as MHW +8.3 %; and as HW +5.6 %) and a longer snout (EN +10.7 %). Taxon 2 has a wider snout (IN +7.5 %) (Fig. 6).

**Figure 7.**
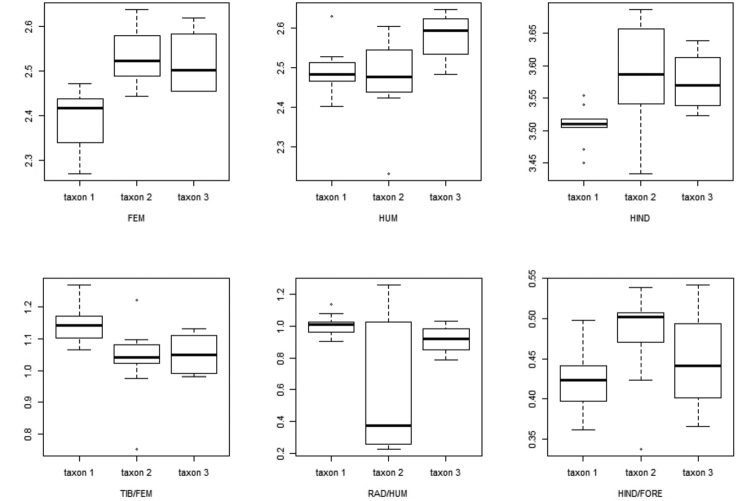
Boxplot of the most differing characters related to limb values between taxon 1, taxon 2, and taxon 3. Characters were corrected to SVL and log–transformed. For abbreviations see Materials and methods.

Regarding the limb data, FEM was 12.7 % longer on taxon 2, as well as the overall hind-limb length (HIND +7.9 %) and the ratio of HIND to FORE (+5.6 %). On the contrary, the ratios of tibia to femur (TIB/FEM +10.2 %) and radius to humerus (RAD/HUM +26.2 %) were larger in taxon 1 (Fig. 7).

**Table 5.** Results from the MANOVA of the log–transformed ratio of characters to SVL of males. The variation was analyzed between the populations from Son La and Thanh Hoa provinces (taxon 1; *N* = 9) and the populations from Phu Tho and Hoa Binh provinces (taxon 2; *N* = 10), and between these (jointly referred to as Tylototriton cf. asperrimus) and the population from Lai Chau Province (taxon 3; in the comparison based on head and dorsum related data: *N_T._*_cf._*_asperrimus_* = 19 and *N*_taxon3_ = 3; and in the comparison of limb related data, *N_T._*_cf._*_asperrimus_* = 21 and *N*_taxon3_ = 4). *F*: *F*–test; DF: degrees of freedom; *P*: *p*–value. For abbreviations of characters see Materials and methods. In **Bold** significant results.

	taxon 1 × taxon 2			T. cf. asperrimus × taxon 3		
	*F*	DF	*P*	*F*	DF	*P*
MHW	17.62	17	**< 0.001**	0.08	20	0.79
HW	7.48	17	**0.01**	52.48	20	**< 0.001**
EN	4.85	17	**0.04**	7.52	20	**0.01**
HL	0.76	17	0.4	0.09	20	0.77
IN	4.56	17	**0.05**	0.09	20	0.77
LJL	2.51	17	0.13	2.22	20	0.15
RAD/HUM	5.92	17	**0.03**	0.34	23	0.56
FEM	21.13	17	**< 0.001**	1.10	23	0.30
TIB/FEM	7.07	17	**0.02**	0.42	23	0.52
HIND	7.47	17	**0.01**	0.43	23	0.52
HIND/FORE	4.95	17	**0.04**	0.03	23	0.87
HUM	0.14	17	0.72	4.66	23	**0.04**
RAD	0.17	17	0.7	<0.001	23	0.98
TIB	1.99	17	0.18	0.83	23	0.78
FORE	0.01	17	0.91	1.87	23	0.19
TL	1.10	17	0.31	0.07	20	0.79
TH	0.35	17	0.56	0.71	20	0.41
TL.TH	0.25	17	0.63	0.56	20	0.46
L5N	0.84	17	0.37	12.43	20	**< 0.01**
WVr	1.61	17	0.22	5.02	20	**0.04**
TkL	0.90	17	0.36	3.74	20	0.07

Comparison of taxon 3 from Lai Chau Province with taxon 1 and taxon 2

This analysis is only based on males. Absolute measures and ratios of species’ morphological traits corrected by snout vent length are shown in Table 4. All three taxa had similar measures for SVL, TL, and TH. The narrowest head was recorded in taxon 3 (HW 15.29 ± 1.04; 19.86 ± 0.95 in taxon 1; 18.75 ± 0.99 in taxon 2) and its maximum values were still below the minima recorded for taxon 1 and taxon 2 (max HW = 16.40 in taxon 3; min HW = 18.44 in taxon 1 and = 16.98 in taxon 2). The snout length was longer in taxon 3 (EN 6.77 ± 0.78), than in taxon 1 (6.16 ± 0.68) or taxon 2 (5.50 ± 0.37). HUM was longer in taxon 3 (13.89 ± 1.69) and showed a maximum range (11.99 to 16.57) not repeated in taxon 1 (11.04 to 13.89) nor in taxon 2 (9.30 to 13.54). Consequently, the sizes of the fore-limbs were also longer in taxon 3 (35.67 ± 2.13, range = 33.88 - 38.9) than in taxon 1 (33.67 ± 0.89, range = 32.43–35.35) and in taxon 2 (33.68 ± 2.11, range = 30.36 - 36.97). In taxon 3 the width of the vertebral cord (WVr 3.53 ± 0.25) and the length of the rib nodules (L5N 3.31 ± 0.87) were wider than in taxon 1 (3.06 ± 0.37, and 2.67 ±0.39, respectively) and taxon 2 (2.90 ± 0.37, and 2.91 ±0.41, respectively). Trunk length, on the other hand was shorter in taxon 3 (TkL 66.22 ± 3.86), than in taxon 1 (70.28 ± 3.41) or taxon 2 (68.78 ± 1.24) (Table 4).

The data set of head and dorsal morphological traits was based on 19 observations of taxon 1 and taxon 2 together and three observations of taxon 3 from Lai Chau. A PCA identified five principal components (PCs) which together explained 84 % of the morphological variation (cumulative explanation of the first 3 PCs = 66 %; of the first 4 PCAs = 75 %). The first two PCs accounted for 48 % of the variation graphically showing a clear separation of the two clades (Fig. 5B). HW, EN, WVr, and L5N were identified as the characters differentiating between the species (MANOVA: *F*_1, 20_ = 20.52, *p* < 0.001) (Table 5). Head width (HW) was 21 % smaller in taxon 3 than in taxon 1 and taxon 2 (*F*_2, 19_ = 36.79, *p* < 0.001), and the size of the rib nodules (L5N) was 15 % longer in taxon 3 than in taxon 1 and taxon 2 (*F*_2, 19_ = 6.59, *p* < 0.01). The two remaining characters were only different between taxon 3 and taxon 2. Both the snout length (EN) and the width of the vertebral cord (WVr) were longer in taxon 3 than in taxon 2, by 17 % (*F*_2, 19_ = 7.21, *p* < 0.01) and 16 % (*F*_2, 19_ = 3.45, *p* < 0.05), respectively (Fig. 6).

The limb data included 21 observations of taxon 1 and taxon 2 together and four of taxon 3 and resulted in a PCA with three PCs explaining 88 % of the variation. The overall MANOVA (*F*_1, 23_ = 1.92, *p* = 0.13) was not significantly different between both lineages (Fig. 5C).

Macroclimatic comparison

Our data show that *T. asperrimus* in Guangxi, China experiences the lowest temperatures during the coldest months (3–6 °C) than any of the remaining three taxa in North Vietnam (12 °C). This species also shows the highest amount of precipitation during the coldest (169–233 mm vs. 38–80 mm for the remaining three taxa) and driest (170–180 mm vs. 38–80 mm for the three remaining taxa) quarter of the year, as well as in the driest month (26–44 mm vs. 4–10 mm for the three remaining taxa) (Table 6).

**Table 6.** Bioclimatic conditions at the species records. Abbreviations: Annual Mean Temperature BIO1, Mean Diurnal Range BIO2, Isothermality BIO3, Temperature Seasonality BIO4, Max Temperature of Warmest Month BIO5, Min Temperature of Coldest Month BIO6, Temperature Annual Range BIO7, Mean Temperature of Wettest Quarter BIO8, Mean Temperature of Driest Quarter BIO9, Mean Temperature of Warmest Quarter BIO10, Mean Temperature of Coldest Quarter BIO11, Annual Precipitation BIO12, Precipitation of Wettest Month BIO13, Precipitation of Driest Month BIO14, Precipitation Seasonality BIO15, Precipitation of Wettest Quarter BIO16, Precipitation of Driest Quarter BIO17, Precipitation of Warmest Quarter BIO18, and Precipitation of Coldest Quarter BIO19.

Variables	Unit	taxon 1	taxon 1	taxon 2	taxon 2	taxon 3	*T. asperrimus*	*T. asperrimus*
BIO1	°C	20.7	20.4	20.4	19.9	19.3	16.8	16.9
BIO2	°C	5.3	5.5	6.1	6.0	6.6	7.4	5.9
BIO3	°C	37.4	38.1	39.7	42.0	45.7	32.0	32.5
BIO4	°C	1.0	1.0	1.0	0.9	0.8	2.0	1.5
BIO5	°C	26.3	26.1	27.1	26.1	26.1	26.0	23.9
BIO6	°C	12.1	11.5	11.7	11.7	11.6	3.0	5.7
BIO7	°C	14.2	14.6	15.4	14.4	14.5	23.0	18.2
BIO8	°C	23.6	23.0	22.8	22.2	20.4	22.1	20.9
BIO9	°C	16.6	16.3	16.5	16.0	15.9	9.5	10.8
BIO10	°C	23.5	23.2	22.3	22.4	21.4	22.7	21.2
BIO11	°C	16.6	16.3	16.3	16.0	15.9	8.5	10.8
BIO12	mm	1884.2	1624.6	1648.3	1603.5	1843.7	1703.7	1558.2
BIO13	mm	379.8	351.2	324.4	373.0	421.6	316.5	335.5
BIO14	mm	7.3	8.2	4.3	6.8	9.6	43.5	25.5
BIO15	mm	92.2	88.6	89.0	93.9	85.8	59.2	74.1
BIO16	mm	984.9	888.1	821.4	910.9	1041.8	784.6	813.5
BIO17	mm	42.6	56.4	45.8	37.8	80.4	189.2	169.6
BIO18	mm	986.2	843.3	403.8	856.8	424.9	390.7	738.1
BIO19	mm	42.6	56.4	43.7	37.8	80.4	233.0	169.6

### Integrative taxonomy

Genetic and morphological differences found in this study support the taxonomic separation between T. cf. asperrimus from Vietnam and *T. asperrimussensu stricto* (from China), thus confirming the distinctness of the Vietnamese clade. Furthermore, we uncovered genetic and morphological variations within the Vietnamese T. cf. asperrimus clade. However, based on our current knowledge these should be evaluated with caution regarding taxon 1 and taxon 2. Therefore these taxa are treated herein at the subspecies level until further evidence is presented. In addition, due to distinct morphological and molecular divergence, the population from Lai Chau Province was revealed to be distinct at the species level.

## Taxonomic accounts

### Tylototriton pasmansi

Taxon classificationAnimalia

M. Bernardes, M. D. Le, T. Q. Nguyen, C. T. Pham, A. V. Pham, T.T. Nguyen & T. Ziegler sp. nov.

A6ADDF55-CA52-51B4-B0D3-52F4FA5FEE8A

http://zoobank.org/3B901B94-4741-40BD-BDC1-75086A06A8FA

Figures 8 , 9

Tylototriton taxon 2 (this study).
T. vietnamensis (referring to the population from Phu Tho Province): Nguyen et al. 2009, page 327.
T. asperrimus (referring to the population from Hoa Binh Province): Yuan et al. 2011, page 583; Nishikawa et al. 2013b, page 39; Luu et al. 2014, page 55.
T. cf. asperrimus (1) (referring to the population from Hoa Binh Province): Phimmachak et al. 2015a, page 293.
T. cf. asperrimus “Lao Cai/Hoa Binh” (referring to the populations from Lac Son, Hoa Binh): Hernandez 2016, page 254.
T. cf. asperrimus “northern Vietnam” (referring to the populations from Lai Chau, Lao Cai, Hoa Binh, and Phu Tho) Hernandez 2018, page 80.

#### Holotype.

IEBR 4466, adult male, collected in Phu Canh Nature Reserve, Da Bac District, Hoa Binh Province, on 11 June 2016 by H. N. Ngo et al.

#### Paratypes.

Four adult males, same data as the holotype: IEBR 4467–IEBR 4470; two adult males collected from Xuan Son National Park, Tan Son District, Phu Tho Province, unknown collector: IEBR 4322 and IEBR 4323; four adult males collected from Xuan Son National Park, Tan Son District, Phu Tho Province, on 7 July 2016 by T. D. Le: IEBR 4320, IEBR 4321, IEBR 4500 and IEBR 4501. One adult female collected from Thuong Tien Nature Reserve (Cot Ca forest, Quy Hoa Commune), Lac Son District, Hoa Binh Province at 720 m elevation on 24 July 2009 by V. Q. Luu: VFU A.2009.8.

**Figure 8.**
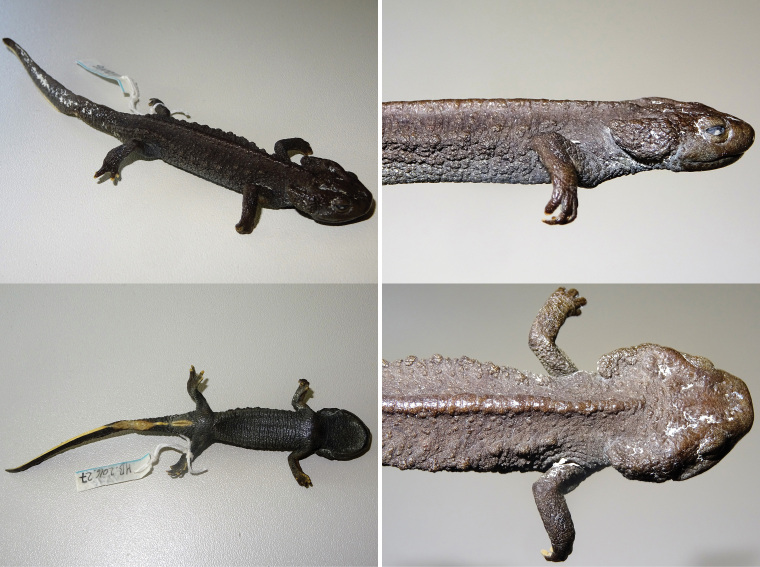
*Tylototriton pasmansi* sp. nov. (holotype). In sequence: dorsal view; ventral view; lateral view; and detail of dorsal view of the head. Photographs T. Ziegler.

#### Etymology.

The species is named after Prof. Dr. Frank Pasmans, Ghent University (Belgium), who has made considerable and path-breaking contributions in the field of infectious diseases driven amphibian declines.

#### Diagnosis.

The new species is diagnosed by the following combination of characters: head slightly longer than wide; snout truncate in dorsal view and slightly angular in profile; relative wide distance between the eyes; distinct mid-dorsal ridge on head; tips of fingers reaching the eye when foreleg adpressed along head; labial and gular folds present; rib nodules distinct and varying from pointy to more rounded; glandular vertebral ridge high, slightly rough and segmented; dorsal skin more granulose than ventral skin; and skin in middle of abdomen with smooth tubercles shaped like transverse wrinkles.

#### Description of holotype.

Habitus moderately slender; head broader than body, slightly longer than wide, depressed and slightly oblique in profile; snout wider than long (IN > EN), truncate in dorsal view, slightly angular shaped in profile and protruding beyond lower jaw; nostrils close to snout tip and slightly visible from above; labial fold slightly evident; dorsolateral bony ridges on head prominent, moderately protruding, from above eye to above anterior end of parotoid, posterior ends relatively thick and scrolled inside; mid-dorsal ridge on head distinct and thin; parotoids enlarged, projecting backwards; ventral skin with tubercles shaped like transverse wrinkles; gular fold weak; glandular vertebral ridge high, slightly rough and segmented, anteriorly thinner, extending from top of head to base of tail, separated from mid-dorsal ridge, with slight scoliosis at height of anterior limbs; number of trunk vertebras around 13; rib nodules distinct, rounded and small, with slightly bigger sizes reached at mid-trunk; tips of fore- and hind limbs touch when adpressed along body; tips of fingers reaching eye when foreleg laid forward; one toe missing on right hind-limb; and tail laterally compressed, thin and tip acuminated.

#### Color of holotype.

**Figure 9.**
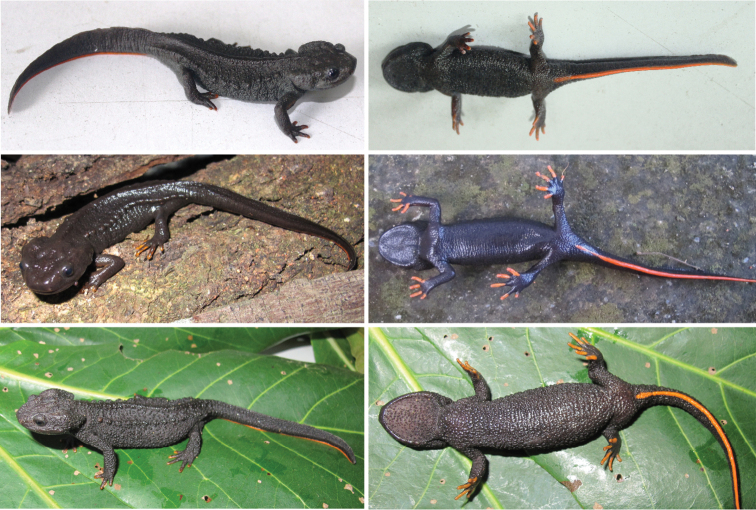
Dorsal and ventral views of the new taxa in life. Top: *Tylototriton pasmansi* sp. nov. (holotype); Center: *Tylototriton pasmansi obsti* ssp. nov. (holotype); Bottom: *Tylototriton sparreboomi* sp. nov. Photographs: C. T. Pham (upper row) and A. V. Pham (central and lower rows).

In preservative, the overall dorsal coloration faded dark grayish green, the ventral coloration dark brown, with faded yellow markings on vent, ventral margin of tail, tip of fingers and toes, and part of palms. For color in life see Fig. 9.

#### Measurements of holotype (in mm).

SVL 64.16; MHW 16.07; HW 11.87; HL 17.67; PL 9.61; PH 4.50; EL 3.06; EN 3.69; IN 5.55; IE 8.11; LJL 10.8; UEL 4.52; HUM 5.97; RAD 13.51; FEM 7.44; TIB 13.70; FORE 19.48; HIND 21.14; TL 53.91; TH 7.78; ClL 8.13; ClW 4.37; WVr 2.18; L5W 2.31; AG 27.36; and TkL 44.00.

#### Variation.

Paratypes from Hoa Binh Province are very similar to the holotype. Paratypes from Phu Tho seem to present a stouter habitus, more distinct middorsal ridge but slightly less protruding dorsolateral ridges on head and slightly enlarged round rib nodules. The variation of the morphological characters in males is summarized in Table 4 and the additional measurements of one female can be found in Table 3.

#### Comparisons.

*Tylototriton pasmansi* sp. nov. differs from other related species of *Tylototriton* as follows: from *T. anhuiensis* by distinctly separated rib nodules (versus continuous nodule-like warts in *T. anhuiensis*); from *T. asperrimus* by a wider (versus shorter) distance between the eyes, tips of fingers reaching eye (versus nostril) when foreleg laid forward, and head slightly longer than wide (versus wider than long in *T. asperrimus* according to Nishikawa et al. 2013b; Sparreboom 2014; Hernandez 2016), however, the female holotype shows similar head proportions–see Discussion); from *T. broadoridgus* by a head longer than wide (versus equally long and wide), wider (versus shorter) distance between eyes, presence (versus absence) of gular fold, smoother (versus extremely rough) skin on ventral side shaped like transverse wrinkles (versus rounded shaped, uniform to dorsal side), distinctly separated rib nodules (versus continuous nodule-like warts), and narrower vertebral ridge (versus broader in *T. broadoridgus*); from *T. hainanensis* by the head being slightly longer than wide (versus much wider than long), and a snout truncate in dorsal view (versus rounded in *T. hainanensis*); from *T. liuyangensis* by a wider (versus shorter) distance between eyes, distinctly separated rib nodules (versus continuous nodule-like warts), and ventral side skin shaped like transverse wrinkles (versus covered by warts in *T. liuyangensis*); from *T. notialis* by a broader (versus narrower) head, a slightly angular (versus rounded) shaped snout in profile, longer (versus shorter) hind-limbs, and higher tail (versus thinner tail in *T. notialis*); from *T. panhai* by wider (versus shorter) distance between the eyes, presence (versus absence) of labial fold, distinct (versus absent) middorsal ridge on head, and dorsal color uniformly dark (versus dorsal color with characteristic colorful markings in *T. panhai*); from *T. vietnamensis* by round to pointy (versus slightly flattened) rib nodules, presence (versus absence) of gular fold, and high vertebral ridge (versus low vertebral ridge in *T. vietnamensis*); from *T. wenxianensis* by a truncate snout in dorsal view (versus round), wider (versus shorter) distance between the eyes, distinctly separated rib nodules (versus continuous nodule-like warts), presence (versus absence) of gular fold, smoother (versus extremely rough) skin on ventral side shaped like transverse wrinkles (versus rounded shaped and uniform to dorsal side), and colored marking on ventral slit (versus black colored ventral slit in *T. wenxianensis*); and from *T. ziegleri* by head slightly longer than wide (versus wider than long), smaller (versus enlarged knob-like) rib nodules, dispersed granules (versus more granulose) on dorsal skin and vertebral ridge slightly less (versus more) segmented and glandular.

The morphological comparison resulting from the measurements performed on the two females of *T. pasmansi* and *T. asperrimussensu stricto* (Table 3) showed that the first one presents: wider (versus narrower) and longer (versus less long) head, longer (versus shorter) lower jaw length, wider (versus shorter) distance between the eyes, as well as higher values for all of the remaining head features, with the exception of snout length and head width, which were, in these cases, higher in *T. asperrimus*. *T. pasmansi* additionally presents longer and higher (versus shorter and thinner) tail, bigger (versus smaller) cloacal muscles, wider (versus narrower) vertebral ridge, slightly bigger (versus smaller) rib nodules and shorter trunk length (versus longer trunk length in *T. asperrimus*).

#### Distribution.

Phu Canh Nature Reserve, Da Bac District and Thuong Tien Nature Reserve, Lac Son District in Hoa Binh Province, and Xuan Son National Park, Tan Son District, Phu Tho Province, Vietnam (Fig. 2).

#### Natural history.

Based on remote sensing information the species is known from sites with an annual mean temperature of 20.4 to 20.7 °C, ranging from 11.5 to 26.3 °C during the year. Annual precipitation is about 1624 to 1884 mm ranging throughout the year from 7.3 to 379.8 mm. Further bioclimatic information is provided in Table 6.

### Tylototriton pasmansi obsti

Taxon classificationAnimalia

M. Bernardes, M. D. Le, T. Q. Nguyen, C.T. Pham, A. V. Pham, T.T. Nguyen & T. Ziegler ssp. nov.

59184B47-F6BD-51F7-A6E6-21356C91016A

http://zoobank.org/7A124E44-4F79-499F-AFD3-5B429D1AB4FB

Figures 9 , 10

Tylototriton taxon 1 (this study).

#### Holotype.

IEBR 4471, adult male, collected in Xuan Nha Nature Reserve, Van Ho District, Son La Province, at an elevation of 1090 m a.s.l., on 15 June 2016 by A. V. Pham and N. B. Sung.

#### Paratypes.

Eight adult males, the same data as the holotype: IEBR 4472–4475, TBU 11–14; two adult males collected at elevation of 950 m a.s.l. in Xuan Lien Nature Reserve, Vin Village, Bat Mot Commune, Thuong Xuan District, Thanh Hoa Province, and in July 2015 by T. S. Nguyen: IEBR 4318 and IEBR 4319.

#### Etymology.

The new subspecies is named after Prof. Fritz-Jürgen Obst, the former herpetologist and director of the Museum für Tierkunde Dresden, Germany, as well as passionate *Tylototriton* keeper, who passed away on the 10 June 2018.

#### Diagnosis.

The new subspecies is diagnosed from the nominotypic subspecies *Tylototriton pasmansi pasmansi* by the following combination of characters: a wider head (both as head width and maximum head width), versus narrower head; a longer and narrower snout, versus shorter and wider snout; a shorter femur and associated hind-limb lengths, versus longer femur and longer hind-limbs length; less overall concentration of warts and small granules on skin, versus overall skin more granulose; and skin on lateral body with apparently same concentration of warts than dorsal side, versus higher concentration of warts on ventral side of the body than on dorsum in *T. p. pasmansi*.

#### Description of holotype.

Habitus moderately stout; head broader than body, slightly longer than wide, depressed and slightly oblique in profile; snout wider than long (IN > EN), truncate in dorsal view, slightly angular shaped in profile and protruding beyond lower jaw; nostrils close to snout tip and not visible from above; labial fold slightly evident; dorsolateral bony ridges on head prominent, moderately protruding, from above eye to above anterior end of parotoid, posterior ends thin and scrolled inside; distinct middorsal ridge on head; parotoids enlarged, projecting backwards; dorsal skin granulose; skin on lateral body and between axilla-groin smooth, with no obvious presence of small glands; throat skin visibly more rough than in between axilla-groin region; gular fold present; glandular vertebral ridge high, slightly rough and segmented, anteriorly thinner, extending from top of head to base of tail, separated from middorsal ridge; number of trunk vertebrae 12; rib nodules distinct, rounded and pointy, with similar sizes throughout their length; fingers from fore- and hind limbs overlap when adpressed along body; tips of fingers reaching eye when adpressed along head; and tail laterally compressed, thin and tip acuminated.

**Figure 10.**
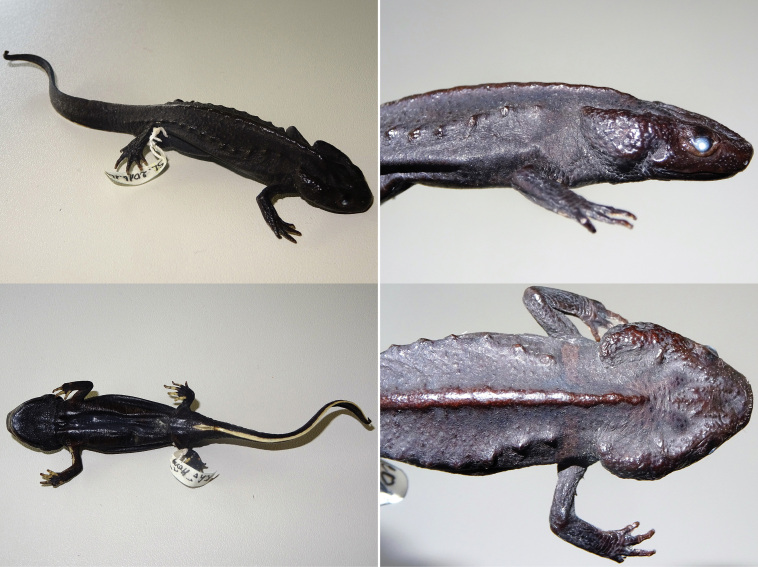
*Tylototriton pasmansi obsti* ssp. nov. (holotype) In sequence: dorsal view; ventral view; lateral view; and detail of dorsal view of the head. Photographs T. Ziegler.

#### Color of holotype.

In preservative, with overall dark brown to blackish with faded yellow markings in vent margin, ventral tail fin, and tips of fingers and toes. For color in life see Fig. 9.

#### Measurements of holotype (in mm).

SVL 67.95; MHW 18.1; HW 12.53; HL 19.44; PL 10.4; PH 5.5; EL 3.4; EN 3.99; IN 5.44; IE 8.64; LJL 12.11; UEL 5.07; HUM 8.12; RAD 14.43; FEM 7.05; TIB 15.75; FORE 22.55; HIND 22.80; TL 60.71; TH 8.46; ClL 8.86; ClW 5.88; WVr 2.18; L5W 2.12; AG 30.57; and TkL 46.48.

#### Variation (based on preserved paratypes).

Some paratypes also show slightly bigger and rounded rib nodules, an overall more granulose skin, and faded yellow coloration on: anterior upper arms (like mating pads), posterior end of parotoids and first rib nodules. The remaining characters were similar to the holotype. Further measurements are summarized in Table 4.

#### Comparisons.

In addition to the diagnostic characteristics already mentioned above, *Tylototriton pasmansi obsti* ssp. nov. differs from *T. p. pasmansi* by having a moderately stout habitus (versus moderately slender, when excluding the population from Phu Tho), nostrils usually not visible (versus usually visible) from dorsal view, usually thinner (versus usually thicker) posterior end of the dorsolateral bony ridges on head, gular fold more evident (versus weaker), rib nodules with similar sizes throughout their length (versus with slightly bigger sizes at mid-trunk), and rib nodules sometimes pointy (versus rounded in *T. p. pasmansi*).

#### Distribution.

Xuan Nha Nature Reserve, Van Ho District, Son La Province and Xuan Lien Nature Reserve, Thuong Xuan District, Thanh Hoa Province, Vietnam (Fig. 2).

#### Natural history.

Specimens were found between 14:00 and 16:00 h inside breeding ponds. The surrounding habitat was characterized by secondary forest of large, medium and small hardwoods mixed with shrubs and vines. Air temperature at the collection time was about 25 to 30 ^o^C and relative humidity was about 75 to 80 %. Based on remote sensing information the species occurs at sites with an annual mean temperature of 19.9 to 20.4 °C, ranging from 11.7 to 27.1 °C during the year. Annual precipitation is about 1603.5 to 1648.3 mm with yearly variations from 4.3 to 373.0 mm monthly. Further bioclimatic information is provided in Table 6.

### Tylototriton sparreboomi

Taxon classificationAnimalia

M. Bernardes, M. D. Le, T. Q. Nguyen, C. T. Pham, A. V. Pham, T.T. Nguyen & T. Ziegler sp. nov.

37553400-6E3E-558F-B0B7-515FC0FE43A0

http://zoobank.org/4599D131-7C89-4D62-B43A-15E24C6473B9

Figures 9 , 11

Tylototriton taxon 3 (this study).
T. sp.: Laking et al. 2017, page 2.
T. verrucosus : Orlov et al. 2002, page 101; van Dijk et al. 2009, page 1; Nguyen et al. 2009, page 329.
T. cf. asperrimus “North Vietnam”: Hernandez 2018, page 80.

#### Holotype.

IEBR 4476, adult male, collected in Sa De Phin Commune, Sin Ho District, Lai Chau Province, Vietnam, at an elevation of 1670 m a.s.l., in May 2015 by A. V. Pham and M. A. Vang.

#### Paratypes.

Two adult males, same data as the holotype: IEBR 4477 and TBU 10; two adult males, collector unknown: IEBR 4478 and IEBR 4479.

#### Etymology.

The specific epithet is dedicated to late Prof. Dr. Max Sparreboom, who has made great contributions to the understanding of Urodela.

#### Diagnosis.

The new species is distinguished from other species of the genus by the following combination of characters: head longer than wide; snout truncate in dorsal view; tips of fingers reaching nostril when foreleg is laid forward; skin tubercles on ventral side shaped like transverse wrinkles; rib nodules distinct and round; vertebral ridge segmented, high and relatively wide; relatively wide distance between the eyes; and gular and labial folds present.

#### Description of holotype.

Habitus stout; head broader than body, longer than wide, depressed and slightly oblique in profile; snout wider than long (IN > EN), truncate in dorsal view, rounded in profile and protruding beyond lower jaw; nostrils close to snout tip and not visible from above; labial fold slightly evident; dorsolateral bony ridges on head prominent, wide, moderately protruding, from above eye to above anterior end of parotoid, posterior ends slightly scrolled inside; middorsal ridge on head almost indistinct; parotoids enlarged, projecting backwards; ventral skin smoother than dorsal skin, with tubercles shaped like transverse wrinkles; gular fold weak; glandular vertebral ridge high, wide, smooth and segmented extending from top of head to base of tail, separated from middorsal ridge; number of trunk vertebrae 13; rib nodules distinct and roundish, the third anterior rib nodule on right side is located below the second nodule and the fourth nodule seems to not be associated with the fourth vertebra, nodules appear knob-like anteriorly, becoming smaller posteriorly; tips of fore- and hind limbs overlap when adpressed along body; tips of fingers reaching nostril when foreleg laid forward; and tail laterally compressed, thin and tip acuminated.

#### Color of holotype.

In preservative, with an overall faded dark brown coloration, with faded yellow markings on vent, ventral margin of tail, tips of fingers and toes, and part of palms. For color in life see Fig. 9.

#### Measurements of holotype (in mm).

SVL 68.71; MHW 17.60; HW 9.85; HL 19.95; PL 10.18; PH 6.12; EL 3.43; EN 4.43; IN 6.26; IE 9.04; LJL 10.74; UEL 4.74; HUM 9.27; RAD 15.86; FEM 8.77; TIB 16.03; TL 59.70; TH 8.57; ClL 9.66; WVr 2.37; L5W 3.04; AG 30.99; and TkL 42.72.

#### Variation.

TBU 10 (in worse preserved condition) presents rib-nodules thinner than holotype, glandular vertebral ridge more tubercular, and tail tip slightly rounded. The remaining characters were similar to the holotype in morphology. For detailed measurements see Table 4.

#### Comparisons.

*Tylototriton sparreboomi* sp. nov. differs from other related species of *Tylototriton* as follows: from *T. anhuiensis* by distinctly separated rib nodules (versus continuous nodule-like warts in *T. anhuiensis*); from *T. asperrimus* by a head longer than wide (versus wider than long in *T. asperrimus* according to Nishikawa et al. 2013b; Sparreboom 2014; Hernandez 2016), however the female holotype shows similar head proportions–see Discussion); from *T. broadoridgus* by head slightly longer than wide (versus equally long and wide), presence of skin on ventral side shaped like transverse wrinkles (versus covered with round shaped tubercles, like the dorsal side), distinctly separated rib nodules (versus continuous nodule-like warts), and narrower vertebral ridge (versus wider vertebral ridge in *T. broadoridgus*); from *T. hainanensis* by a head longer than wide (versus much wider than long), tips of fingers reaching nostril (versus eyes) when foreleg is laid forward, and a snout truncate in dorsal view (versus rounded in *T. hainanensis*); from *T. liuyangensis* by a wider (versus shorter) distance between eyes, distinctly separated rib nodules (versus continuous nodule-like warts), and lateral skin shaped like transverse wrinkles (versus covered by warts in *T. liuyangensis*); from *T. notialis* by a broader (versus narrower) head, longer (versus shorter) hind-limbs, and higher tail (versus thinner tail in *T. notialis*); from *T. panhai* by wider (versus shorter) distance between the eyes, presence (versus absence) of labial fold, and overall dorsal coloration mostly dark (versus with presence of characteristic dorsal colorful markings in *T. panhai*); from *T. pasmansi* sensu lato by a narrower (versus wider) head, slightly wider (versus slightly narrower) distance between the eyes, tips of fingers reaching nostril (versus eye) when foreleg laid forward, longer (versus shorter) humerus length, and slightly enlarged round-like rib nodules (versus slightly smaller, pointy to rounded rib nodules in *T. pasmansi* sensu lato); from *T. p. pasmansi* by a longer (versus shorter) length between eye and nostril and wider (versus narrower) vertebral ridge in *T. p. pasmansi*; from *T. pasmansi obsti* by a longer (versus shorter) femur length; from *T. vietnamensis* by a moderately stout (versus slender) habitus, presence (versus absence) of gular fold, and round (versus slightly flattened) rib nodules and high vertebral ridge (versus low vertebral ridge in *T. vietnamensis*); from *T. wenxianensis* by a truncate (versus more rounded) snout in dorsal view, wider (versus narrower) distance between the eyes, distinctly separated rib nodules (versus continuous nodule-like warts), smoother (versus extremely rough) skin on ventral side shaped like transverse wrinkles (versus rounded shaped and uniform to dorsal side), and colored marking on ventral slit (versus black colored ventral slit in *T. wenxianensis*); from *T. ziegleri* by a head longer than wide (versus wider than long), rounded but smaller (versus enlarged knob-like) rib nodules, and distinctly segmented vertebral ridge (versus even more segmented vertebral ridge in *T. ziegleri*).

**Figure 11.**
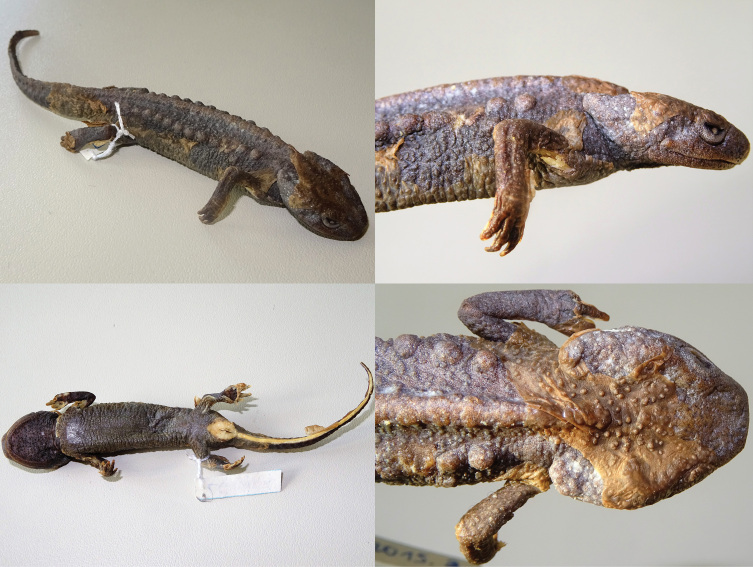
*Tylototriton sparreboomi* sp. nov. (holotype). In sequence: dorsal view; ventral view; lateral view; and detail of dorsal view of the head. Photographs T. Ziegler.

#### Distribution.

Known only from the type locality in Lai Chau Province, northern Vietnam (Fig. 2).

#### Natural history.

Specimens were found in water between 9:00 and 16:30 h in ponds. The surrounding habitat was secondary forest of large, medium and small hardwoods mixed with shrubs and vines. Air temperature at the sites was 23 to 27 °C and relative humidity was 80 to 85%. Based on remote sensing information, the species occurs at sites with an annual mean temperature of 19.3 °C, ranging from 11.6 to 26.1 °C during the year. Annual precipitation is about 1843.7 mm with yearly variations from 9.6 to 421.6 mm. Further bioclimatic information is provided in Table 6.

## Discussion

Based on examples listed in Table 7, we argue that clear genetic and morphological differences help clarify the taxonomic status of the taxa in question. The genetic differences between *T. pasmansi* and its closest relative *T. asperrimus* (from 3.2 to 3.6 %), between *T. sparreboomi* and *T. asperrimus* (from 4.1 to 4.2 %), and between *T. pasmansi* and *T. sparreboomi* (from 3.6 to 4.5 %) are higher than other minimum genetic distances between species of the genus *Tylototriton* (see *T. shanjing* × *T. verrucosus* × *T. pulcherrimus* × *T. podichthys*, *T. anguliceps* × *T. pulcherrimus*, *T. broadoridgus* × *T. dabienicus*, *T. anhuiensis* × *T. broadoridgus*, and *T. ngarsuensis* × *T. shanorum*).

**Table 7.** Examples of integrative taxonomy in other species of the genus *Tylototriton*. *partial 16S rRNA and COI, and complete tRNA Leu, ND1, ND2, tRNA Ile, tRNA Gln, tRNA Met, tRNA Trp, tRNA Ala, tRNA Asn, tRNA Cys, and tRNA Tyr.

Source	Gene used	Species at stake	Genetic var. (%)	Morphological variation and conclusions
Zhang et al. (2007)	partial cyt b	*T. shanjing* × *T. verrucosus*	mean 1.2; range 0.4–2.6	Morphology not discussed. Conspecificity. Stuart et al. (2010), Nishikawa et al. (2013a) and Nishikawa et al. (2015) point out that only a single, unvouchered sample of *T. verrucosus* was included, and cautiously defended the need for further examinations before taxonomic conclusions.
Khatiwada et al. (2015)	complete ND2 & partial cyt b	*T. shanjing* × *T. verrucosus*	1.9	Size; head proportions; grooves on tail base; coloration. “The topotypic *T. verrucosus* were deeply nested within *T. shanjing*.”
Khatiwada et al. (2015)	complete ND2 & partial cyt b	*T. pulcherrimus* × *T. verrucosus; T. pulcherrimus* × *T. shanjing*	2.1; 2.8	Not further discussed. Treated as separated species.
Nishikawa et al. (2013a)	partial ND2, POMC & Rag 1	*T. shanjing* (Jingdong and Nu Jiang, Yunnan, China)	mean 1.4; range 0.2–2.1	Morphology not discussed. Treated as intraspecific variation.
Nishikawa et al. (2013a)	partial ND2, POMC & Rag 1	*T. shanjing* (from above) × *T. pulcherrimus*	mean 2.6; range 2.5–2.8	Morphology not discussed. Considered conspecific due to small genetic differences.
Nishikawa et al. (2015)	partial ND2	*T. yangi* × *T. daweishanensis*	mean 0.4; range 0.2–0.5	Coloration. Considered conspecific due to small genetic differences.
Khatiwada et al. (2015)	complete ND2 & partial cyt b	*T. yangi* × *T. daweishanensis*	mean 0.7	Not discussed. Treated as separate species.
Phimmachak et al. (2015a)	*	*T. anguliceps* × *T. pulcherrimus*	mean 3.1; range 2.8–3.4	Morphology not discussed. Treated as separate species and used as example for low genetic divergence within species of the genus.
Phimmachak et al. (2015a)	*	*T. podichthys* (description) × *T. pulcherrimus*	mean 2.9; range 2.5–3.4	Morphology not discussed. The new species formed a unique clade within an unresolved polytomy containing *T. verrucosus*, *T. shanjing*, and *T. pulcherrimus*.
Phimmachak et al. (2015a)	*	*T. podichthys* (description) × *T. shanjing*	mean 3.4; range 2.1–4.6	Ridge on midline of crown; coloration. Treated as separate species.
Le et al. (2015)	partial ND2	*T. podichthys* (Xam Neua, Laos) × *T. shanjing*	0.4	When the paper was published, *T. podichthys* was not yet described. The authors referred to this population as *T. verrucosus* from Laos, which formed a clade with *T. shanjing*, *T. pulcherrimus*, and *T. verrucosus* from the type locality.
Phimmachak et al. (2015a)	*	*T. podichthys* (description) × *T. verrucosus*	mean 3.1; range 2.1–4.4	Ridge on midline of crown; coloration; skin on cranial crest; orientation of parotoids. Separate species.
Shen et al. (2012)	complete ND2	*T. broadoridgus* (description) × *T. wenxianensis*	mean 3.9; range 3.8–4	Dorsal ridge; height of tail; presence of genital papillae; form of rib warts. Treated as separate species.
Shen et al. (2012)	complete ND2	*T. broadoridgus* (description) × *T. dabienicus*	mean 3.5; range 3.4–3.5	Inferred morphological differences. Separate species.
Nishikawa et al. (2013b)	partial ND2	*T. broadoridgus* × *T. dabienicus*	mean 3.3; range 3.3–3.4	Morphology not examined. Treated as separate species and used as example for low genetic divergence within species of the genus.
Nishikawa et al. (2013a)	partial ND2	*T. broadoridgus* × *T. dabienicus*	3.3	Morphology not examined. The authors suspect conspecificity.
Khatiwada et al. (2015)	complete ND2 & partial cyt b	*T. broadoridgus* × *T. dabienicus*	3.4	Not discussed. Treated as separate species.
Stuart et al. (2010)	* (except COI)	*T. notialis* (description) × *T. hainanensis*	range 3.7–3.8	Form of rib warts. Treated as separate species.
Nishikawa et al. (2013b)	partial ND2	*T. notialis* (type from Laos) × *T. notialis* (Nghe An, Vietnam)	2.8	Coloration. Considered conspecific.
Nishikawa et al. (2013b)	partial ND2	*T. ziegleri* (description)	mean 1.7; range 0.1–2.8	Treated as intraspecific variation.
Nishikawa et al. (2013b)	partial ND2	*T. asperrimus* (China) × *T. asperrimus* (Thuong Tien, Hoa Binh, Vietnam)	mean 2.7; range 0.1–3.4	Morphology not examined. Considered conspecific by Nishikawa et al. (2013b); separate species, Tylototriton pasmansi, this study.
Qian et al. (2017)	ND1, ND2 & cyt b	*T. ziegleri* x *T. vietnamensis*	2.3	Known morphological differences based on Nishikawa et al. (2013b): ridges on head; skin; vertebral ridge; form of rib warts; tail size and height. Treated as separate species.
Qian et al. (2017)	ND1, ND2 & cyt b	*T. anhuiensis* (description) × *T. broadoridgus*	3.2	Dorsal ridge width; head proportions. Treated as separate species.
Grismer et al. (2018)	ND2	*T. ngarsuensis* (description) × *T. shanorum*	range 3.0–3.4	Size; head length; rib nodules; dorsal ridge; parotoid position; coloration. Treated as separate species.

It becomes apparent that these genetic differences, accompanied by clear morphological disparities, warrant taxonomic revision. The shape of the head of the holotype of *T. asperrimus* (a female) is slightly longer than wide, but evidence from literature, likely based on males, supports a head morphology being (slightly) wider than long in this species (Nishikawa et al. 2013b; Sparreboom 2014; Hernandez 2016). In the diagnostic comparisons above, we followed the major consensus found in literature and used head shape as a diagnostic characteristic to separate *T. asperrimus*, *T. pasmansi*, and *T. sparreboomi*. Seglie et al. (2010) found differences between the head shape of males and females in *T. himalayanus*. In *T. asperrimus* it is currently not clear if this change of head proportions is an exceptional occurrence or a feature related to sexual dimorphism. Until this issue is clarified, the diagnostic feature should be employed with reservations. The dorsal coloration described herein (as it is usually the case within this subgenus) should also be used cautiously as a diagnostic characteristic. Additional work might uncover intra-specific color variations, as in *T. notialis* (Nishikawa et al. 2013b), and *T. ziegleri* (Hernandez 2016).

Two taxa, *T. p. pasmansi* and *T. pasmansi obsti*, are herein cautiously described as subspecies, since their genetic divergences are lower (from 2.5 to 3.1 %) and morphological differences are more subtle. In this case, additional surveys and genetic study (e.g., microsatellites, nuclear DNA analysis) should follow to provide a more complete taxonomic evaluation of these taxa. These two subspecies appear to be separated by the Da River [Black River] (see Fig. 2). Since the Da River is the largest river in northwestern Vietnam, it may serve as a physical barrier restricting the gene flow between populations. Recently, Hernandez et al. (2018) assessed the patterns of macro-ecological niche differentiation in Asian crocodile newts. The authors suggested that both niche conservatism as well as niche differentiation can be detected. Many species occupy forested habitats at higher elevations, which are characterized by cooler and moister micro-habitats compared to lowland and open areas. Although our low sample size prevents detailed niche assessments for the new taxa, the overall niche structure within the genus suggests that the taxa are restricted to micro-climatic pockets in mountainous areas. Hence, not only the river systems represent likely barriers for gene flow, but also unsuitable micro-climatic conditions might limit dispersals in the region.

Wang et al. (2018) hypothesized that *Tylototritonsensu lato* originated from the ancestral area consisting of northern Indochina Peninsula and southern Yunnan Province during the middle Miocene, approximately 15 MYA. This period coincided with the warming Miocene climate optimum (Zachos et al. 2001; Böhme 2003). It is also evident that the speciation rates of the group have slowed down significantly from the beginning of the Pleistocene, probably due to the global cooling during this epoch (Wang et al. 2018). However, an important period between just before the Pliocene, around 6 MYA, and the Pleistocene, approximately 2.5 MYA, when a majority of speciation events within the group occurred (see Fig. 4 in Wang et al. 2018), has not been discussed in previous studies. During the Pliocene, the global temperature was about 2–5 °C higher than that of the pre-industrial time (Zachos et al. 2001; Ravelo et al. 2004; Salzmann et al. 2011). Warmer climates, which have been shown to promote diversification rates in different animal groups, including ants, mammals, plethodontid salamanders, and softshell turtles, possibly through increased dispersal rates (Moreau et al. 2006; Smith et al. 2006; Vieites et al. 2007; Le et al. 2014), are also likely to influence the evolution of the Knobby Salamanders in Asia. Our study also suggests that the two new species emerged during this period, further supporting the higher rate of diversification of *Tylototriton* in the Pliocene.

Wang et al. (2018) and also Zaw et al. (2019) did not find support for the monophyly of *T. asperrimus*. Their phylogeny placed the population from Thuong Tien, Hoa Binh (*T. p. pasmansi*) as conspecific to the *T. asperrimus* form from China (identified together as *T. asperrimus* lineage 1 in both studies). These studies also misidentified the population from Xinyi County, Guangdong Province, China as *T. asperrimus* lineage 2, although this population forms a sister clade to the topotypic *T. hainanensis* from Hainan Island. The population from Baise, which according to Hernandez (2018) is distributed in Napo and Jingxi counties, China, was also recovered as a sister taxon to *T. hainanensis* in previous studies (Yuan et al. 2011; Phimmachak et al. 2015a; Hernandez 2016). Moreover, the studies by Wang et al. (2018) and Zaw et al. (2019) did not support the monophyly of both, *T. wenxianensis* and *T. dabienicus* species complexes. The *T. wenxianensis* complex appears to consist of at least two undescribed lineages, one in Libo county, Guizhou Province and the other in Wufeng county, Hubei Province, while in the *T. dabienicus* complex both studies apparently missed the description of *T. anhuiensis* by Qian et al. (2017), and as a result, continue to identify the population from Yuexi, Anhui as an unknown lineage sister to *T. dabienicus*.

The so far undescribed population from Lao Cai Province, Vietnam has been successively attributed to a number of species: T. cf. vietnamensis (Böhme et al. 2005), *T. asperrimus* (Nguyen et al. 2009), purportedly *T. ziegleri* (Nishikawa et al. 2013b), and T. cf. asperrimus “northern Vietnam” (Hernandez 2018). Phimmachak et al. (2015a), using a comprehensive phylogenetic sample, defended the conspecificity of the population from Lao Cai with the population from Lac Son, Hoa Binh Province, which we herein describe as *T. p. pasmansi*.

Based on these new developments we suspect *T. asperrimus* (type) to be endemic to Guangxi Province, in China. It is distributed in Jinxiu Yao Autonomous County in Mt. Dayao (including Dayaoshan Nature Reserve [Hernandez 2016] and in Bainiu locality [Hernandez 2018]) and in Mt. Xianglu (also Xianglushan) (Yang et al. 2014); in Longsheng County (Shen et al. 2012); Ziyuan County in Mt. Miao‘er (Hernandez 2018); in Huanjiang County including the Mulun Nature Reserve (Qin et al. 2012); and in Tian’e County (Hernandez 2018). According to Hernandez (2016 and 2018) the species has a wide distribution in fragmented mountain areas from northern through central and southern Guangxi. The population from E’huangzhang Mts., Yangchun County, Guangdong is shown as a sister to topotypic *T. asperrimus* (Hernandez 2016 and Hernandez 2018), but still awaits proper taxonomic allocation.

Given the high demand of *Tylototriton* species in the international trade, and the persistent evidence of a high poaching rate (Gong and Mu 2008; Nishikawa et al. 2014; Phimmachak et al. 2015b; Rowley et al. 2016; Bernardes et al. 2017; Grismer et al. 2018) we decided to follow Hou et al. (2014) and cautiously omit the exact locations in this manuscript.

## Outlook

This study increases the currently known number of *Tylototriton* species from northern Vietnam, from four (*T. anguliceps*, *T. notialis*, *T. vietnamensis*, and *T. ziegleri*) to six and one subspecies, by discovering *T. sparreboomi*, *T. p. pasmansi* and *T. pasmansi obsti*. It also further affirms that this region supports the highest diversity within this genus (Nishikawa et al. 2013b).

The taxonomic separation of a single widespread species into multiple small-ranged taxa in turn has important implications for the conservation status of the original species (Köhler et al. 2005; Stuart et al. 2006). We therefore recommend a re-assessment of the outdated Near Threatened status of *T. asperrimussensu lato* (van Dijk et al. 2008) to reflect taxonomic revisions and increasingly severe threats from international trade and habitat loss, which have taken place over the last decade.

## Supplementary Material

XML Treatment for Tylototriton pasmansi

XML Treatment for Tylototriton pasmansi obsti

XML Treatment for Tylototriton sparreboomi

## Supplementary materials

Supplementary material 1 Regression of each morphological character to its respective snout-vent length value for taxon 1 and taxon 2 Data type measurement https://binary.pensoft.net/file/412509This dataset is made available under the Open Database License (http://opendatacommons.org/licenses/odbl/1.0/). The Open Database License (ODbL) is a license agreement intended to allow users to freely share, modify, and use this Dataset while maintaining this same freedom for others, provided that the original source and author(s) are credited.Marta Bernardes, Minh Duc Le, Truong Quang Nguyen, Cuong The Pham, Anh Van Pham, Tao Thien Nguyen, Dennis Rödder, Michael Bonkowski, Thomas Ziegler

Supplementary material 2 Time-calibrated tree of *Tylototriton* based on ND2 sequences. The values indicate the split time (in million years ago) calculated by BEAST 1.8.0. Data type measurement https://binary.pensoft.net/file/412510This dataset is made available under the Open Database License (http://opendatacommons.org/licenses/odbl/1.0/). The Open Database License (ODbL) is a license agreement intended to allow users to freely share, modify, and use this Dataset while maintaining this same freedom for others, provided that the original source and author(s) are credited.Marta Bernardes, Minh Duc Le, Truong Quang Nguyen, Cuong The Pham, Anh Van Pham, Tao Thien Nguyen, Dennis Rödder, Michael Bonkowski, Thomas Ziegler

## References

[B1] Bain RH Truong NQ (2004) Herpetofaunal diversity of Ha Giang Province in northeastern Vietnam, with descriptions of two new species. American Museum Novitates 3453(2004): 1–42. 10.1206/0003-0082(2004)453<0001:HDOHGP>2.0.CO;2

[B2] Bernardes M Pham CT Nguyen TQ Le MD Bonkowski M Ziegler T (2017) Comparative morphometrics and ecology of a newly discovered population of *Tylototriton vietnamensis* from northeastern Vietnam including remarks on species conservation. Salamandra 53(3): 451–457.

[B3] Böhme M (2003) The Miocene Climate Optimum: evidence from ectothermic vertebrates of Central Europe. Palaeogeography, Palaeoclimatology, Palaeoecology 195: 389–401. 10.1016/S0031-0182(03)00367-5

[B4] Böhme W Schöttler T Nguyen TQ Köhler J (2005) A new species of salamander, genus *Tylototriton* (Urodela: Salamandridae) from northern Vietnam. Salamandra 41(4): 215–220.

[B5] Deblauwe V Droissart V Bose R Sonké B Blach-Overgaard A Svenning JC Wieringa JJ Ramesh BR Stévart T Covereur TLP (2016) Remotely sensed temperature and precipitation data improve species distribution modelling in the tropics. Global Ecology and Biogeography 25(4): 443–454. 10.1111/geb.12426

[B6] Drummond AJ Ho SYW Phillips MJ Rambaut A (2006) Relaxed phylogenetics and dating with confidence. PloS Biology 4(5): 699–710. 10.1371/journal.pbio.0040088 PMC1395354 16683862

[B7] Drummond AJ Rambaut A (2007) BEAST: Bayesian evolutionary analysis by sampling trees. BMC Evolutionary Biology 7(1): 214 10.1186/1471-2148-7-214 17996036 PMC2247476

[B8] Dubois A Raffaëlli J (2009) A new ergotaxonomy of the family Salamandridae Goldfuss, 1820 (Amphibia, Urodela). Alytes 26(1–4): 1–85.

[B9] Fei L Ye C Yang R (1984) A new species and a new subspecies of the genus *Tylototriton* (Caudata: Salamandridae). Acta Zoologica Sinica 30(1): 85–91. [in Chinese]

[B10] Felsenstein (1985) Confidence limits on phylogenies: an approach using the bootstrap. Evolution 39(4): 783–791. 10.1111/j.1558-5646.1985.tb00420.x 28561359

[B11] Gong D-J Mu M (2008) Behavioral observations and descriptions of the endangered knobby newt *Tylototriton wenxianensis* and their application in conservation. Asiatic Herpetological Research 11(2008): 31–38.

[B12] Grismer LL Junior PLW Quah ESH Thura MK Espinoza RE Grismer MS Murdoch ML Lin A (2018) A new species of Crocodile Newt *Tylototriton* (Caudata: Salamandridae) from Shan State, Myanmar (Burma). Zootaxa 4500(4): 553–573. 10.11646/zootaxa.4500.4.5 30486050

[B13] Grismer LL Wood PL Quah ESH Thura MK Espinosa RE Murdoch ML (2019) A new species of crocodile newt *Tylototriton* (Caudata: Salamandridae) from northern Myanmar (Burma). Journal of Natural History 53(7–8): 475–495. 10.1080/00222933.2019.1587534

[B14] Hernandez A (2016) Crocodile Newts, The Primitive Salamandridae from Asia (Genera *Echinotriton* and *Tylototriton*). Edition Chimaira, Frankfurt, 415 pp.

[B15] Hernandez A (2018) Ecological affinities of *Tylototriton asperrimus* Unterstein, 1930 (Amphibia, Caudata: Salamandridae) at Bainiu, Dayaoshan, Guangxi Province, China with an emphasis on its cryptic diversity. Bulletin de la Société Herpétologique de France 166(2018): 79–82.

[B16] Hernandez A Escoriza D Hou M (2018) Patterns of niche diversification in south-east Asian crocodile newts. Zoologischer Anzeiger 276(2018): 86–93. 10.1016/j.jcz.2018.06.001

[B17] Hernandez A Escoriza D Pomchote P Mian H (2019) New localities for *Tylototriton uyenoi*, *T. panhai* and *T. anguliceps* in Thailand with remarks on the southernmost distribution of the genus. The Herpetological Bulletin 147(2019): 15–18. 10.33256/hb147.1518

[B18] Hillis DM Bull JJ (1993) An empirical test of the bootstrap as a method for assessing confidence in phylogenetic analysis. Systematic Biology 42(2): 182–192. 10.1093/sysbio/42.2.182

[B19] Hou M Wu Y Yang K Zheng S Yuan Z Li P (2014) A missing geographic link in the distribution of the genus *Echinotriton* (Caudata: Salamandridae) with description of a new species from Southern China. Zootaxa 3895(1): 89–102. 10.11646/zootaxa.3895.1.5 25543556

[B20] IUCN SSC Amphibian Specialist Group (2016) *Tylototriton vietnamensis* The IUCN Red List of Threatened Species 2016: e.T135868A88920562. 10.2305/IUCN.UK.2016-3.RLTS.T135868A88920562.en

[B21] IUCN SSC Amphibian Specialist Group (2017) *Tylototriton ziegleri* The IUCN Red List of Threatened Species 2017: e.T47144899A47144905. 10.2305/IUCN.UK.2017-2.RLTS.T47144899A47144905.en

[B22] Jiang Y Wei Z Han F Ni Q Yao Y Xu H Li Y Rao D Zhang M (2017) The complete mitogenome sequence of *Tylototriton ziegleri* (Amphibia: Caudata). Conservation Genetics Resources 9(3): 503–506. 10.1007/s12686-017-0710-8

[B23] Khatiwada JR Wang B Ghimire S Vasudevan K Paudel S Jiang JP (2015) A new species of the genus *Tylototriton* (Amphibia: Urodela: Salamandridae) from eastern Himalaya. Asian Herpetological Research 6(4): 245–256.

[B24] Köhler J Vieites DR Bonett RM García FH Glaw F Steinke D Vences M (2005) New amphibians and global conservation: A boost in species discoveries in a highly endangered vertebrate group. Bioscience 55(8): 693–696. 10.1641/0006-3568(2005)055[0693:NAAGCA]2.0.CO;2

[B25] Laking AE Ngo HN Pasmans F Martel A Nguyen TT (2017) *Batrachochytrium salamandrivorans* is the predominant chytrid fungus in Vietnamese salamanders. Scientific Reports 7(44443): 1–5. 10.1038/srep44443 28287614 PMC5347381

[B26] Le M Duong HT Dinh LD Nguyen TQ Pritchard PCH McCormack T (2014) A phylogeny of softshell turtles (Testudines: Trionychidae) with reference to the taxonomic status of the critically endangered, giant softshell turtle, *Rafetus swinhoei*. Organisms, Diversity & Evolution 14(3): 279–293. 10.1007/s13127-014-0169-3

[B27] Le DT Nguyen TT Nishikawa K Nguyen SLH Pham AV Matsui M Bernardes M Nguyen TQ (2015) A new species of *Tylototriton* Anderson, 1871 (Amphibia: Salamandridae) from Northern Indochina. Current Herpetology 34(1): 38–50. 10.5358/hsj.34.38

[B28] Luu VQ Le CX Do HQ Hoang TT Nguyen TT Bonkowski M Ziegler T (2014) New records of amphibians from Thuong Tien Nature Reserve, Hoa Binh Province, Vietnam. Herpetological Notes 7(2014): 51–58.

[B29] Moreau CS Bell CD Vila R Archibald SB Pierce NE (2006) Phylogeny of the ants: diversification in the age of angiosperms. Science 312(5770): 101–104. 10.1126/science.1124891 16601190

[B30] Nguyen TQ Nguyen VS Ho LT Le QK Nguyen TT (2009) Phylogenetic relationships and taxonomic review of the family Salamandridae (Amphibia: Caudata) from Vietnam (in Vietnamese). Vietnam Journal of Biotechnology 7(3): 325–333.

[B31] Nishikawa K Khonsue W Pomchote P Matsui M (2013a) Two new species of *Tylototriton* from Thailand (Amphibia: Urodela: Salamandridae). Zootaxa 3737(3): 261–279. 10.11646/zootaxa.3737.3.5 25112754

[B32] Nishikawa K Matsui M Nguyen TT (2013b) A new species of *Tylototriton* from Northern Vietnam (Amphibia: Urodela: Salamandridae). Current Herpetology 32(1): 34–49. 10.5358/hsj.32.34

[B33] Nishikawa K Matsui M Rao D (2014) A new species of *Tylototriton* (Amphibia: Urodela: Salamandridae) from Central Myanmar. Natural History Bulletin of the Siam Society 60(1): 9–22.

[B34] Nishikawa K Rao D Matsui M Eto K (2015) Taxonomic relationship between *Tylototriton daweishanensis* Zhao, Rao, Liu, Li & Yuan, 2012 and *T. yangi* Hou, Li & Lu, 2012. Current Herpetology 34(1): 67–74. 10.5358/hsj.34.67

[B35] Oksanen J Blanchet F Kindt R Legendre P Minchin P O’Hara R Simpson G Solymos P Stevens M Wagner H (2015) Vegan: community ecology. R package version 2.2-1. R Project for Statistical Computing, Vienna, Austria.

[B36] Orlov N Murphy RW Ananjeva NB Ryabov SA Cuc HT (2002) Herpetofauna of Vietnam, a checklist. Part I Amphibia. Russian Journal of Herpetology 9(2): 81–104.

[B37] Phimmachak S Aowphol A Stuart BL (2015a) Morphological and molecular variation in *Tylototriton* (Caudata: Salamandridae) in Laos, with description of a new species. Zootaxa 4006(2): 285–310. 10.11646/zootaxa.4006.2.3 26623768

[B38] Phimmachak S Stuart BL Aowphol A (2015b) Ecology and natural history of the knobby newt *Tylototriton podichthys* (Caudata: Salamandridae) in Laos. Raffles Bulletin of Zoology 63(2015): 389–400.

[B39] Posada D Crandall KA (1998) Modeltest: testing the model of DNA substitution. Bioinformatics (Oxford, England) 14(9): 817–818. 10.1093/bioinformatics/14.9.817 9918953

[B40] Qian L Sun X Li J Guo W Pan T Kang X Jiang J Wu J Zhang B (2017) A new species of the genus *Tylototriton* (Amphibia: Urodela: Salamandridae) from the southern Dabie Mountains in Anhui Province. Asian Herpetological Research 8(3): 151–164.

[B41] Qin W Tang X Qin G Wei L (2012) Preliminary survey of *Tylototriton asperrimus* habitat in Mulun Nature Reserve, Guangxi (in Chinese). Sichuan Journal of Zoology 31(2): 303–306.

[B42] Ravelo AC Andreasen DH Lyle M Lyle AO Wara MW (2004) Regional climate shifts caused by gradual global cooling in the Pliocene epoch. Nature 429(6989): 263–267. 10.1038/nature02567 15152244

[B43] Ronquist F Teslenko M van der Mark P Ayres DL Darling A Höhna S Larget B Liu L Suchard MA Huelsenbeck JP (2012) MrBayes 3.2: efficient Bayesian phylogenetic inference and model choice across a large model space. Systematic Biology 61(3): 539–542. 10.1093/sysbio/sys029 22357727 PMC3329765

[B44] Rowley JJL Shepherd CR Stuart BL Nguyen TQ Hoang HD Cutajar TP Wogan GOU Phimmachak S (2016) Estimating the global trade in Southeast Asian newts. Biological Conservation 199(2016): 96–100. 10.1016/j.biocon.2016.05.001

[B45] Salzmann U Williams M Haywood AM Johnson ALA Kender S Zalasiewicz J (2011) Climate and environment of a Pliocene warm world. Palaeogeography, Palaeoclimatology, Palaeocology 309(1–2): 1–8. 10.1016/j.palaeo.2011.05.044

[B46] Seglie D Roy D Giacoma C (2010) Sexual dimorphism and age structure in a population of *Tylototriton verrucosus* (Amphibia: Salamandridae) from the Himalayan Region. Copeia 2010(4): 600–608. 10.1643/CG-08-218

[B47] Shen Y Jiang J Mo X (2012) A new species of the genus *Tylototriton* (Amphibia, Salamandridae) from Hunan, China. Asian Herpetological Research 3(1): 21–30. 10.3724/SP.J.1245.2012.00021

[B48] Simmons JE (2002) Herpetological collecting and collections management. Revised edition. Society for the Study of Amphibians and Reptiles. Herpetological Circular 31: 153 pp.

[B49] Smith T Rose KD Gingerich PD (2006) Rapid Asia-Europe- North America geographic dispersal of earliest Eocene primate *Teilhardina* during the Paleocene-Eocene thermal maximum. Proceedings of the National Academy of Sciences 103(30): 11223–11227. 10.1073/pnas.0511296103 PMC1544069 16847264

[B50] Sparreboom M (2014) Salamanders of the Old World: the salamanders of Europe, Asia and northern Africa. Knnv Publishing, Zeist, Netherlands, 385 pp 10.1163/9789004285620

[B51] Stuart BL Inger RF Voris HK (2006) High level of cryptic species diversity revealed by sympatric lineages of Southeast Asian forest frogs. Biology Letters 2(3): 470–474. 10.1098/rsbl.2006.0505 17148433 PMC1686201

[B52] Stuart BL Phimmachak S Sivongxay N Robichaud WG (2010) A new species in the *Tylototriton asperrimus* group (Caudata: Salamandridae) from central Laos. Zootaxa 2650(2010): 19–32. 10.11646/zootaxa.2650.1.2

[B53] Sun S-J Dai Q Dai Z-X Zhang H-M Gong R-H Du J-F Zou H-S Nie CA (2011) Population resource and habitat selection in summer of Black Knobby Newt (*Tylototriton asperrimus*) in surrounding areas of Houhe National Nature Reserve, Hubei Province, China (in Chinese). Chinese Journal of Ecology 30(11): 2534–2539.

[B54] Swofford DL (2003) PAUP*. Phylogenetic Analysis Using Parsimony (*and Other Methods), version 4.0 b10. Sinauer Associates, Sunderland, Massachusetts.

[B55] Thompson JD Gibson TJ Plewniak F Jeanmougin F Higgins DG (1997) The ClustalX windows interface: Flexible strategies for multiple sequence alignment aided by quality analysis tools. Nucleic Acids Research 25(24): 4876–4882. 10.1093/nar/25.24.4876 9396791 PMC147148

[B56] van Dijk PP Nguyen TQ Wai NLM Ermi Z Shunqing L (2008) *Tylototriton asperrimus* The IUCN Red List of Threatened Species 2008: e.T59482A11932895. 10.2305/IUCN.UK.2008.RLTS.T59482A11932895.en

[B57] van Dijk PP Wogan G Lau MWN Dutta S Shrestha TK Roy D Truong NQ (2009) *Tylototriton verrucosus*. IUCN Red List Threatened Species 2009 e.T59487A11934912.

[B58] van Ende CN (2001) Repeated-measures analysis: growth and other time-dependent measures. In: Scheiner S Gurevitch J (Eds) Design and analysis of ecological experiments.Chapman and Hall, New York, 134–157.

[B59] Vieites DR Min M-S Wake DB (2007) Rapid diversification and dispersal during periods of global warming by plethodontid salamanders. Proceedings of the National Academy of Sciences 104(50): 19903–19907. 10.1073/pnas.0705056104 PMC2148395 18077422

[B60] Wang B Nishikawa K Matsui M Nguyen TQ Xie F Li C Khatiwada JR Zhang B Gong D Mo Y Wei G Chen X Shen Y Yang D Xiong R Jiang J (2018) Phylogenetic surveys on the newt genus *Tylototriton**sensu lato* (Salamandridae, Caudata) reveal cryptic diversity and novel diversification promoted by historical climatic shifts. PeerJ 6(2018): e4384. 10.7717/peerj.4384 PMC5853667 29576937

[B61] Weisrock DW Papenfuss TJ Macey JR Litvinchuk SN Polymeni R Ugurtas IH Zhao E Jowkar H Larson A (2006) A molecular assessment of phylogenetic relationships and lineage accumulation rates within the family Salamandridae (Amphibia, Caudata). Molecular Phylogenetics and Evolution 41(2): 368–383. 10.1016/j.ympev.2006.05.008 16815049

[B62] Yang D Jiang J Shen Y Fei D (2014) A new species of the genus *Tylototriton* (Urodela: Salamandridae) from northeastern Hunan Province, China. Asian Herpetological Research 5(1): 1–11. 10.3724/SP.J.1245.2014.00001

[B63] Yuan Z-Y Jiang K Lü S-Q Yang J-X Nguyen TQ Nguyen TT Jin J-Q Che J (2011) A phylogeny of the *Tylototriton asperrimus* group (Caudata: Salamandridae) based on a mitochondrial study: suggestions for a taxonomic revision. Zoological Research 32(6): 577–84. 22184015 10.3724/SP.J.1141.2011.06577

[B64] Zachos J Pagani M Sloan L Thomas E Billups K (2001) Trends, rhythms, and aberrations in global climate 65Ma to present. Science 292(5517): 686–693. 10.1126/science.1059412 11326091

[B65] Zaw T Lay P Pawangkhanant P Gorin VA Poyarkov Jr NA (2019) A new species of Crocodile Newt, genus *Tylototriton* (Amphibia, Caudata, Salamandridae) from the mountains of Kachin state, northern Myanmar. Zoological Research 40(3): 151–174. 10.24272/j.issn.2095-8137.2019.043 31011130 PMC6591163

[B66] Zhang M-W Rao D-Q Yu G-H Yang J-X (2007) The validity of red knobby newt (*Tylototriton shanjing*) species status based on mitochondrial Cyt b gene. Zoological Research 28(4): 430–436.

