# Supplementary material for: Integrative taxonomy reveals three new taxa within the Tylototriton asperrimus complex (Caudata, Salamandridae) from Vietnam
Source: Zookeys. 2020 May 21;935:121–64. doi: 10.3897/zookeys.935.37138 (PMC7256073; doi:10.3897/zookeys.935.37138)
Supplement: Supplementary material 1 — Regression of each morphological character to its respective snout-vent length value for taxon 1 and taxon 2 [file zookeys-935-121-s001.docx]

**Supplementary material:**

Regression of each morphological character to its respective snout-vent length value for taxon 1 and taxon 2:

Maximum head width


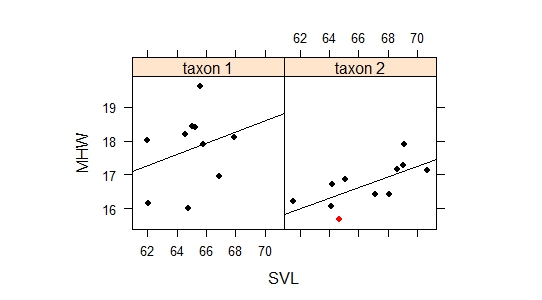


Head length


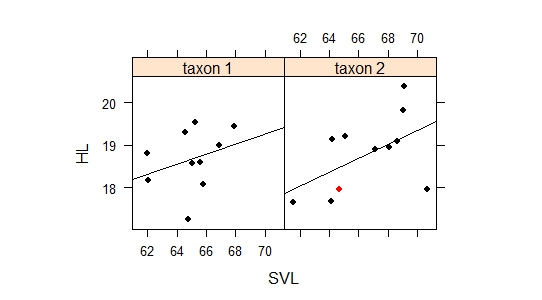


Eye-narial distance


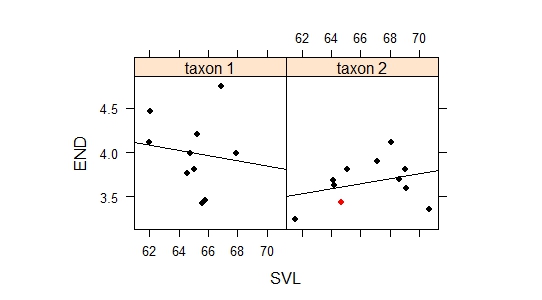


Inter-nariel distance


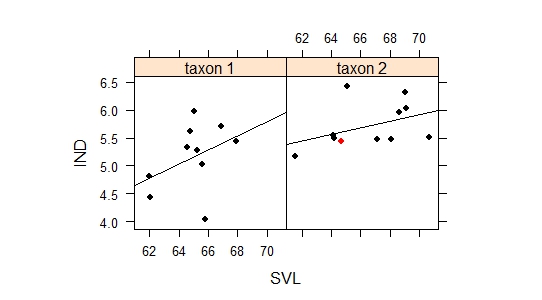


Head width


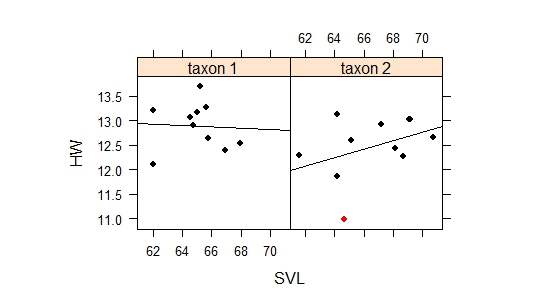


Lower law length


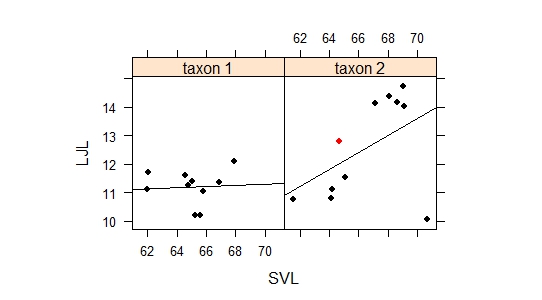


Humerus length


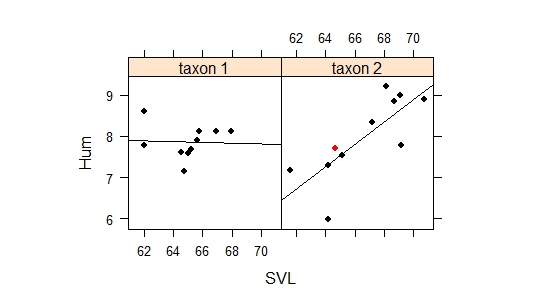


Radius length


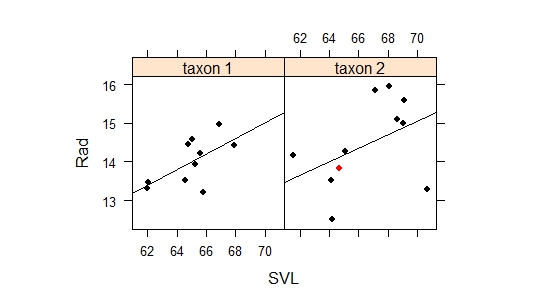


Femur length


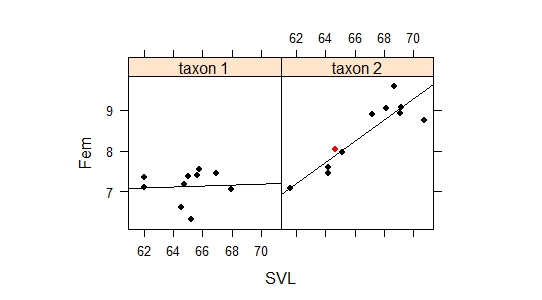


Tibia length


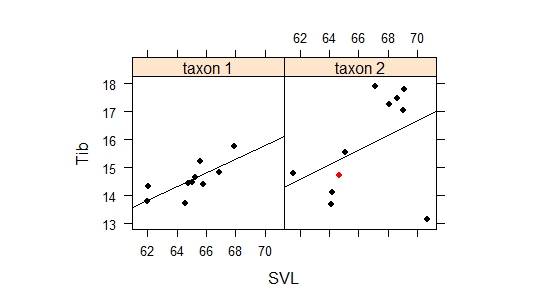


Forelimb length


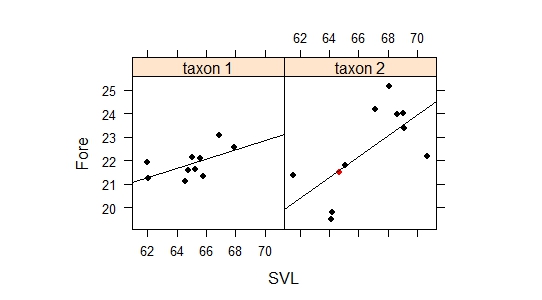


Hind-limb length


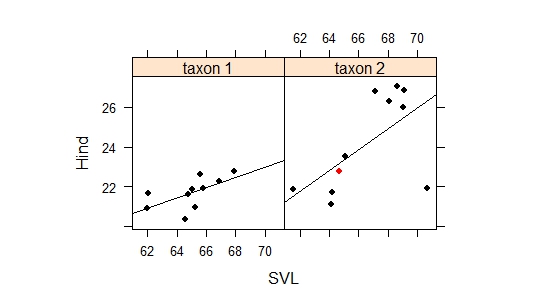


Ratio Hind- /Forelimb


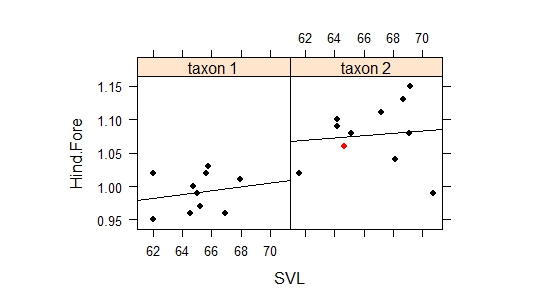


Ratio Radius to Humerus length


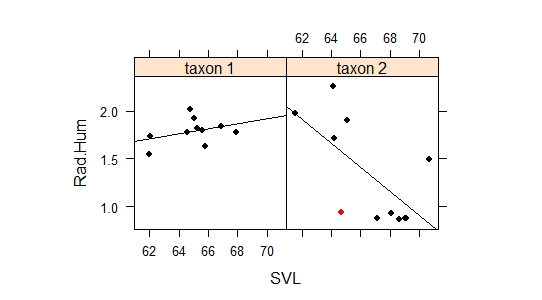


Ratio Tibia to Femur length


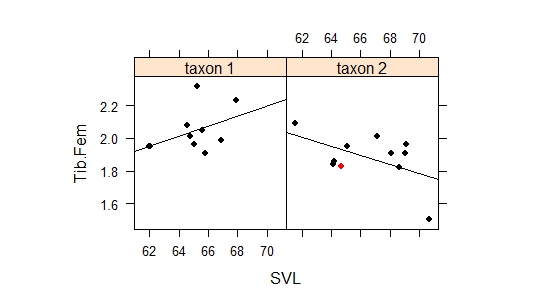


Tail length


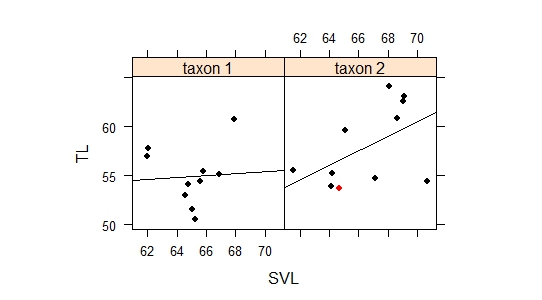


Tail height


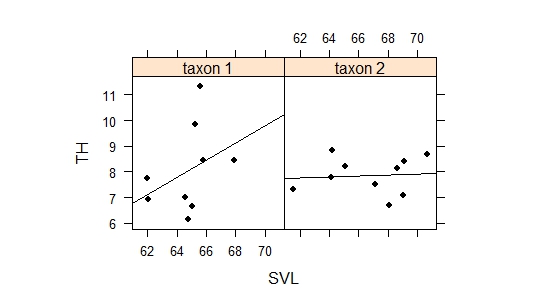


Ratio tail length to tail height


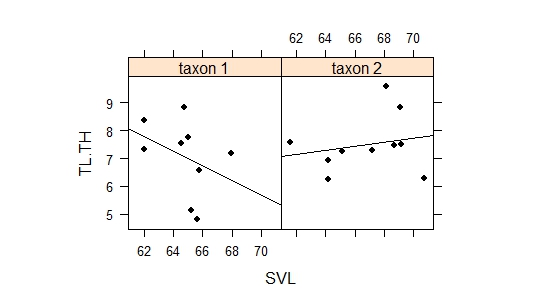


Width of vertebral cord


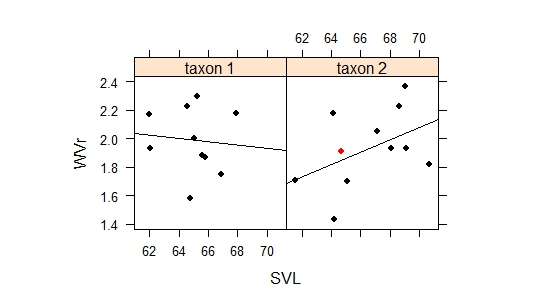


Length dorsal nodule


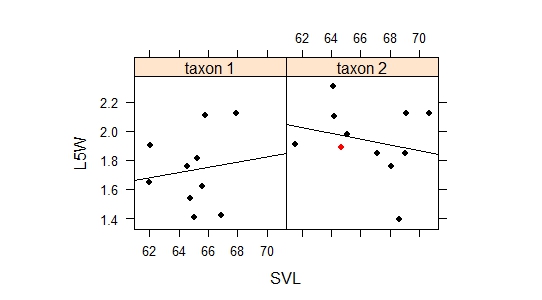


Trunk length


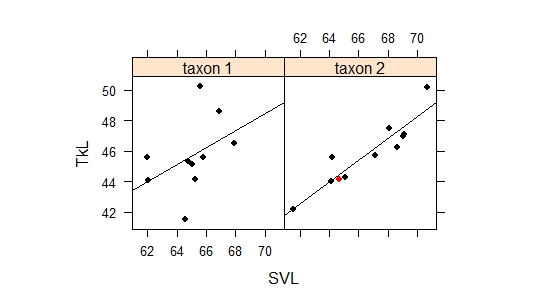


Pearson correlation coefficients (r) and corresponding p-value (p) between morphological traits and SVL. In bolt, statistically significant results.

| **Traits:** | **taxon 1** | | **taxon 2** | |
| --- | --- | --- | --- | --- |
|  | **r** | **p** | **r** | **p** |
| MHW | 0.28 | 0.44 | **0.69** | **0.02** |
| HL | 0.31 | 0.38 | 0.51 | 0.11 |
| END | -0.13 | 0.71 | 0.31 | 0.35 |
| IND | 0.39 | 0.26 | 0.40 | 0.22 |
| HW | -0.05 | 0.88 | 0.39 | 0.24 |
| LJL | 0.05 | 0.89 | 0.47 | 0.14 |
| Hum | -0.04 | 0.92 | **0.77** | **0.01** |
| Rad | **0.62** | **0.05** | 0.43 | 0.19 |
| Fem | 0.05 | 0.88 | **0.90** | **<0.001** |
| Tib | **0.75** | **0.01** | 0.41 | 0.21 |
| Fore | 0.58 | 0.08 | **0.67** | **0.02** |
| Hind | 0.61 | 0.06 | **0.60** | **0.05** |
| Hind/Fore | 0.18 | 0.62 | 0.10 | 0.77 |
| Rad/Hum | 0.36 | 0.31 | **-0.65** | **0.03** |
| Tib/Fem | 0.43 | 0.22 | -0.52 | 0.10 |
| TL | 0.06 | 0.87 | 0.50 | 0.12 |
| TH | 0.37 | 0.33 | 0.08 | 0.83 |
| TL/TH | -0.36 | 0.34 | 0.21 | 0.56 |
| WVr | -0.09 | 0.80 | 0.45 | 0.17 |
| L5W | 0.13 | 0.72 | -0.23 | 0.49 |
| TkL | 0.42 | 0.22 | **0.92** | **<0.001** |
