# Supplementary figures and images for: Integrative taxonomy reveals three new taxa within the Tylototriton asperrimus complex (Caudata, Salamandridae) from Vietnam
Source: Zookeys. 2020 May 21;935:121–64. doi: 10.3897/zookeys.935.37138 (PMC7256073; doi:10.3897/zookeys.935.37138)

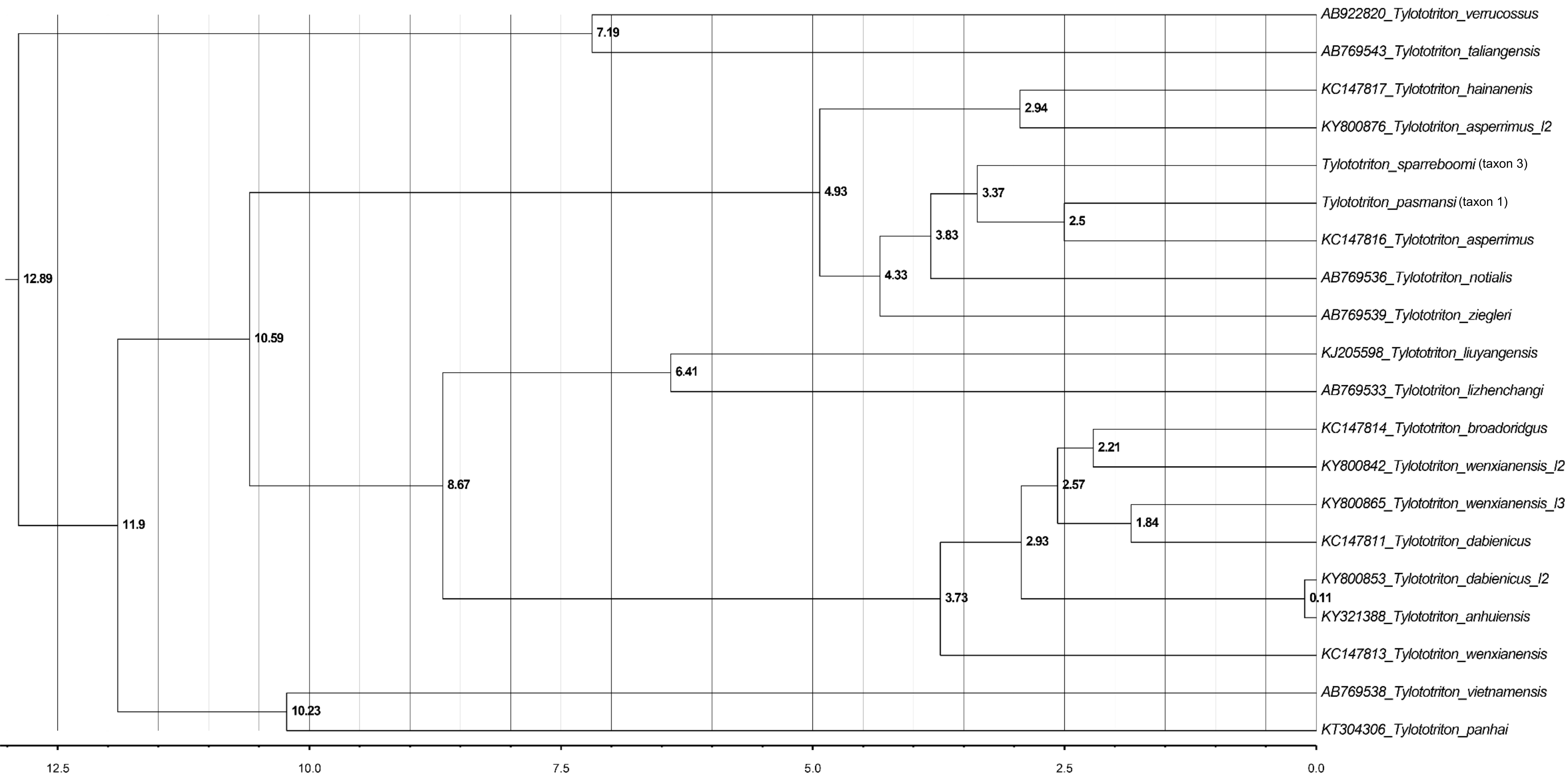

Supplement: Supplementary material 2 — Time-calibrated tree of Tylototriton based on ND2 sequences. The values indicate the split time (in million years ago) calculated by BEAST 1.8.0. [file zookeys-935-121-s002.png]
